# Systematic cortical thickness and curvature patterns in primates

**DOI:** 10.1016/j.neuroimage.2023.120283

**Published:** 2023-07-27

**Authors:** Nagehan Demirci, Mia E. Hoffman, Maria A. Holland

**Affiliations:** a Bioengineering Graduate Program, University of Notre Dame, Notre Dame, IN 46556, USA; b Department of Mechanical Engineering, University of Washington, Seattle, WA 98195, USA; c Department of Aerospace and Mechanical Engineering, University of Notre Dame, Notre Dame, IN 46556, USA

**Keywords:** Cortical thickness, Curvature, Shape index, Cortical folding, Primates, Sulcal depth

## Abstract

Humans are known to have significant and consistent differences in thickness throughout the cortex, with thick outer gyral folds and thin inner sulcal folds. Our previous work has suggested a mechanical basis for this thickness pattern, with the forces generated during cortical folding leading to thick gyri and thin sulci, and shown that cortical thickness varies along a gyral–sulcal spectrum in humans. While other primate species are expected to exhibit similar patterns of cortical thickness, it is currently unknown how these patterns scale across different sizes, forms, and foldedness. Among primates, brains vary enormously from roughly the size of a grape to the size of a grapefruit, and from nearly smooth to dramatically folded; of these, human brains are the largest and most folded. These variations in size and form make comparative neuroanatomy a rich resource for investigating common trends that transcend differences between species. In this study, we examine 12 primate species in order to cover a wide range of sizes and forms, and investigate the scaling of their cortical thickness relative to the surface geometry. The 12 species were selected due to the public availability of either reconstructed surfaces and/or population templates. After obtaining or reconstructing 3D surfaces from publicly available neuroimaging data, we used our surface-based computational pipeline (https://github.com/mholla/curveball) to analyze patterns of cortical thickness and folding with respect to size (total surface area), geometry (i.e. curvature, shape, and sulcal depth), and foldedness (gyrification). In all 12 species, we found consistent cortical thickness variations along a gyral–sulcal spectrum, with convex shapes thicker than concave shapes and saddle shapes in between. Furthermore, we saw an increasing thickness difference between gyri and sulci as brain size increases. Our results suggest a systematic folding mechanism relating local cortical thickness to geometry. Finally, all of our reconstructed surfaces and morphometry data are available for future research in comparative neuroanatomy.

## 1. Introduction

The folded cortical surface of the human brain has attracted the interest of researchers from diverse disciplines for many decades. Some of the earliest histological measurements of cortical thickness revealed consistent patterns — gyri are thicker than sulci (Bok, 1929, 1960; Brodmann, 1908; Brodmann and Gary, 2006; Economo and Triarhou, 2009; Welker, 1990). Korbinian Brodmann (1868–1918), for example, worked extensively on human brain mapping, comparative neuroanatomy, and the evolution of the cortex among different species. He investigated the regional and global cortical thickness variations throughout ontogeny and phylogeny, both within individuals and across species. He observed that homologous regions tend to be thick or thin across species (Brodmann and Gary, 2006). Later, Constantin von Economo (1876–1931) and George Koskinas (1885–1975) substantially improved the measurement of cortical thickness by cutting histological slices of the brain perpendicularly to the axes of gyri and sulci (Economo and Triarhou, 2009). In their data, they noted the gradual decrease of cortical thickness from the top of gyri to lateral walls and to the sulcal valleys (Economo and Triarhou, 2009).

Subsequent researchers attempted to uncover the explanation why. Siegfried Bok (1892–1964) explained the variation in cortical thickness as the consequence of relative volume preservation in cortical layers throughout the folds of the brain (Bok, 1929; Consolini et al., 2022). Much later, Welker (1990) proposed that gyral crowns are thicker than sulcal fundi because of the variations of neuronal differentiation and arborization: the neurons and their neuropils occupy space dispersely and elongate radially in gyri, whereas they reside compactly and elongate longitudinally in sulci (Welker, 1990; Bok, 1929; Cowan, 1979; Razavi et al., 2017). Additional research has shown that there are significantly more neurons in gyri than sulci (Razavi et al., 2017; Hilgetag and Barbas, 2005), and that intracortical axons appear to proliferate more in gyri compared to sulci (Zhang et al., 2017; Razavi et al., 2017; Xu et al., 2010; Chen et al., 2013b). Although these explanations give the impression that consistent variations of cortical thickness are the consequence of neuronal differences between gyri and sulci, they might as well be the cause. As Bok hypothesized, neurons might sense the environmental cues and alter their density, orientation, and shape due to the change of curvature throughout the in-and-out folds of the cortex via mechanosignaling to fulfill the volume-constancy principle (Bok, 1929).

More recently, magnetic resonance (MR) images have allowed for the analysis of cortical thickness *in vivo*. With the advancements in automated neuroimaging pipelines, the complex 3-D morphology of the cortex can be extracted, allowing us to quantify cortical thickness through surface-based (Fischl and Dale, 2000; Dahnke et al., 2013; Kabani et al., 2001; Lerch and Evans, 2005; Hayashi et al., 2021) or voxel-based methods (Scott et al., 2009; Hutton et al., 2008). Cortical thickness estimations from these pipelines have been validated against estimations from histology studies (Cardinale et al., 2014; Rosas et al., 2002) or with other pipelines (Kharabian Masouleh et al., 2020; Velázquez et al., 2021; Tustison et al., 2014), encouraging wide acceptance and usage. Many recent studies utilizing these algorithms have also found consistent gyral–sulcal thickness differences (Holland et al., 2018; Wang et al., 2021; Fischl and Dale, 2000; Zhang et al., 2017; Lin et al., 2021).

While some studies have shown that thickness differences can determine the position of cortical folds (Zhang et al., 2016), our previous work has also shown the opposite: that the buckling of a thin layer on a soft substrate necessarily bifurcates into thick peaks and thin valleys (Holland et al., 2018). In theoretical, computational, and experimental studies, this was shown to be a universal phenomenon of bilayered instabilities, resulting from the physical forces experienced during buckling. (While the stimulus for this buckling is still not completely understood, it is believed to result from residual stresses arising from differential growth in the cortex (Richman et al., 1975; Bayly et al., 2013), likely driven by heterogeneous gene expression in the subplate (Ronan and Fletcher, 2015), potentially alongside tension generated by axon tracts in the underlying white matter (Van Essen, 1997; Xu et al., 2010).) From our previous work, gyral–sulcal thickness differences are predicted to increase with 1) increasing foldedness, 2) increasing cortical thickness, and 3) decreasing gray-white matter stiffness ratio (Holland et al., 2018). However, it is not the case that physical forces alone are responsible for thickness differences. Simulations of cortical folding with preferential growth in gyri turned out to best reproduce the patterns of thickness seen in the brain (Wang et al., 2021), suggesting that increased growth in gyri serves to further exaggerate thickness differences.

In our recent study in humans, we expanded the investigation of cortical thickness, generally studied in a gyral–sulcal binary, to include lateral walls and saddle shapes. We demonstrated a strong correlation between cortical thickness and geometrical shape, with thickness decreasing along a gyral–sulcal spectrum — consistently thickest for convex shapes, thinnest for concave shapes, and in the middle for saddle shapes (Demirci and Holland, 2022). For our shape analysis, we utilized dimensionless shape index, introduced by Koenderink and van Doorn (1992), which varies from −1 to 1. Shape index is a very useful measure in characterizing complex patterns of cortical folds. With a single measure, local shape is conveniently extracted at each point of the cortex. This presents itself as a methodological advantage both in terms of easing morphometric calculations and eliminating the need for segmenting complex structures, for example, sulci fundi and gyral crests. In this study, we follow a similar approach and use shape index as the primary measure of curvature.

Now, we are interested in exploring this finding to see if human brains are an exception, or if the same phenomenon can be observed in other primate species. Primate species brains span a huge range of forms, sizes, and degrees of foldedness. In early development, the cortex is smooth for all species, but at later stages of ontogeny, cortices of different species exhibit different forms and patterns (Welker, 1990; Hayashi et al., 2021; Essen et al., 2019). This variation in size and form among related species offers a rich testing ground for theories of neurodevelopment. Based on our previous work, we hypothesize that the mechanical forces that emerge during the development of the brain, together with other cellular and genetic determinants, lead to this systematic morphological trait of the cortex, and therefore that the distribution of cortical thickness along the gyral–sulcal spectrum will be similar to what we have observed in humans.

To that end, we aim to investigate the relationship between cortical morphology and cortical thickness in a variety of primate species. Additionally, we analyze the changes in cortical morphology with respect to age in several species, to determine if the changes seen in aging humans are present for other primates. However, investigating primate brains is challenging for a number of reasons; including the lack of available imaging data (both in the diversity of species and the number of specimens); the lack of standardization in data acquisition and scanning protocols; and the lack of software for the reconstruction of cortical surfaces.

First of all, it is challenging to obtain images of non-human primate species. It is, fortunately, fairly straightforward to access thousands of human brain MR images and multiple population templates, for example from ABIDE (Autism Brain Imaging Data Exchange) (Di Martino et al., 2014) and the Human Connectome Project (Glasser et al., 2016). However, the availability of non-human primate data is much more limited, particularly for those species who are less commonly used as laboratory research animals. The PRIMatE Resource Exchange (Milham et al., 2018) and the National Chimpanzee Brain Resource (NCBR) (https://www.chimpanzeebrain.org) are both valuable data repositories that share macaque and chimpanzee neuroimaging data, with many specimens available for each species. But for many other species, fewer specimens have been scanned and made available (for example, the marmoset Liu et al., 2021; Hayashi et al., 2021), and for yet others of the 350 extant primate species, no images are publicly available.

Secondly, the diversity of sizes and forms among primate brains has led to a range of neuroimaging protocols and hardware, which are far less standardized than human neuroimaging (Autio et al., 2021; Milham et al., 2022). For example, ultra high field scanners (7T) require narrower bore sizes and constrain the RF coil size. These non-standard RF coils might be susceptible to intensity bias fields and distortion (Autio et al., 2021; Milham et al., 2018). Total head size of the species is another concern for inter-species heterogeneity that requires optimized and adjusted field-of-view to improve spatial resolution. Subject motion also impacts the image quality, with awake subject scanning protocols more prone to motion artifacts and reduced image quality. Additionally, non-standard image resolution and tissue-contrast between white and gray matter complicate important procedures such as brain extraction, image registration, and tissue segmentation. For example, humans are typically scanned with 1 mm isotropic resolution, but for brains of other sizes, the rule is to have an isotropic spatial resolution of half the minimum cortical thickness (Autio et al., 2021).

Finally, the lack of software packages for the reconstruction of cortical surfaces in non-human primates poses a significant challenge. Detailed analysis of cortical morphology requires reconstructed surfaces (both the outer pial surface and, for cortical thickness measurements, the surface at the interface of the white and gray matter). These surfaces must be created via brain extraction and tissue segmentation. Many automated pipelines such as Freesurfer (Fischl, 2012) and BrainVisa (Cointepas et al., 2001), are optimized for humans and often fail to process non-human brains. To address this, researchers have to either adapt existing tools, optimized for human brains, to the analysis of non-human brains, or develop new tools. These attempts have resulted in numerous customized solutions for a single species, with more generalized solutions that are applicable to a range of non-human primate species (Messinger et al., 2021), but these products are still evolving.

Generally, the segmentation approaches being developed fall into one of two main categories: template-based and intensity-based methods. A template-based approach requires a population-averaged, single-animal template which serves as a representative brain of that population, providing a common framework for individual subject scans, which are then aligned to the template through linear and non-linear registration algorithms (Chumchob and Ke, 2009; Zhang et al., 2001). Templates can be very efficient; after aligning the individual image, the remaining steps of skull-stripping and tissue segmentation are more straightforward, minimizing the need for tedious, time-consuming manual interventions. Unfortunately, templates are costly to produce, requiring high-resolution and ideally *in vivo* scans from multiple subjects, and are thus only available for a handful of the species most commonly used in research.

Intensity-based approaches, on the other hand, rely on the intensity contrast between different tissue types to delineate their locations. Although a population template is not required for this approach, multiple parameters need to be optimized for each non-human species, which is done through tedious trial and error. While recent advances have yielded more sensitive segmentation (Gulban et al., 2018), these approaches are not fully automatized.

**Fig. 1.**
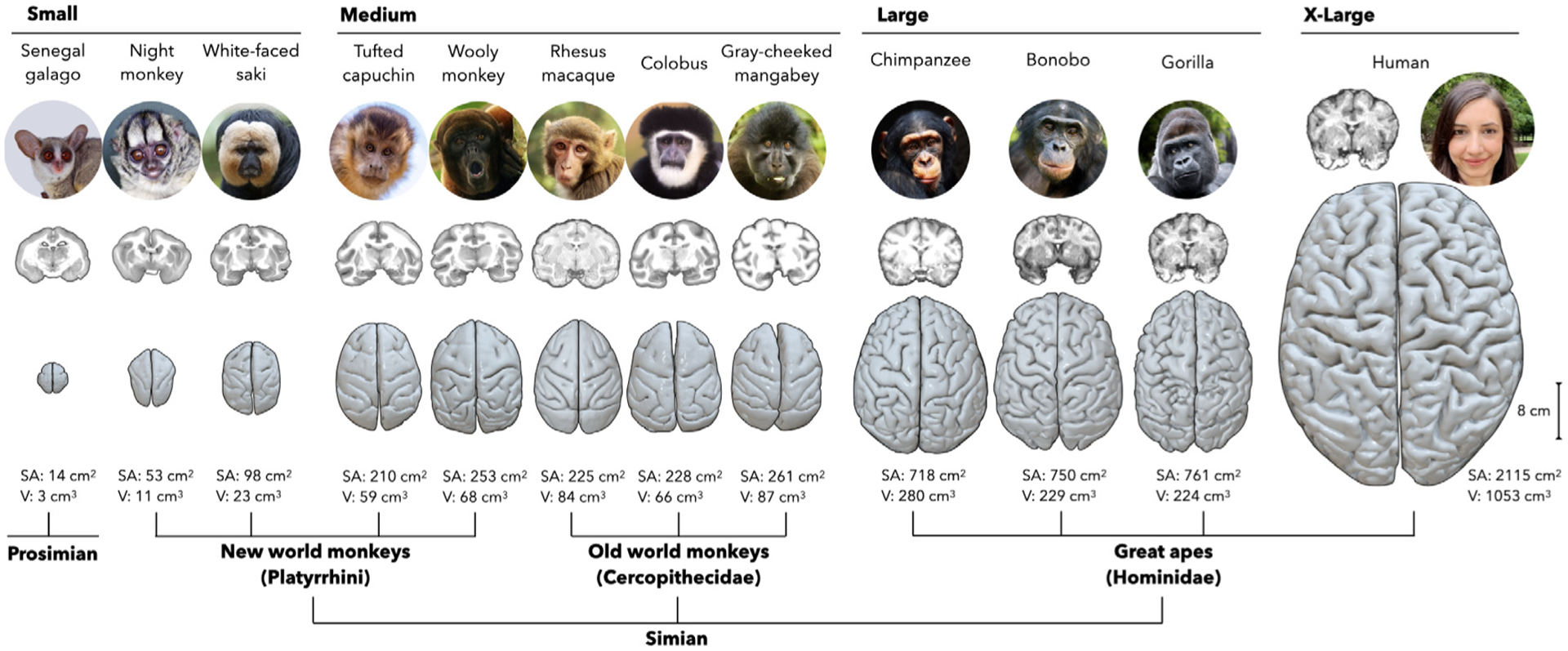
The primate species investigated in this study. Each species is listed by its common name, and is accompanied by a picture and a coronal MR slice, both shown not to scale, and a reconstructed cortical surface, shown to scale. Total surface area (SA) and cerebral volume (V) are listed below. Species are arranged in order of increasing surface area with the exception of the wooly monkey, which is shifted two species to the left to demonstrate the phylogenetic classification. Surface area spans two orders of magnitude, with species distributed unevenly along that axis; therefore, to facilitate analysis, we divided them into four groups (small, medium, large, and x-large) based on natural breaks in the distribution of their surface areas. The night monkey, colobus, and gray-cheeked mangabey images are copyright 2009 Marie Hale (https://flickr.com/photos/15016964@N02/5568808375), 2018 Eric Kilby (https://www.flickr.com/photos/ekilby/26790822947), and 2012 Joe McKenna (https://www.flickr.com/photos/jpmckenna/8183556861), respectively, and are adapted under a Creative Commons Attribution 2.0 Generic License.

Given these challenges, we identified 12 primate species for study (Fig. 1). Their brains range dramatically in size from the smallest, Senegal galago, about the size of a grape, to the largest, humans, about the size of a grapefruit (Fig. 1). We selected these species because they cover a wide range of size and form; represent two suborders (simians and prosimians), seven families, and eleven genera within the primate order (from left to right in Fig. 1: *Galago*, *Aotus*, *Pithecia*, *Sapajus*, *Macaca*, *Colobus*, *Lagothrix*, *Lophocebus*, *Pan*, *Pan*, *Gorilla*, *Homo*); and, conveniently, have either reconstructed surfaces and/or population templates publicly available. Then, by using our surface-based, open-source computational pipeline (Demirci and Holland, 2022) (https://github.com/mholla/curveball), we analyzed the patterns of cortical thickness and folding for each species with respect to size (surface area), geometry (curvature, shape, and sulcal depth), and degree of foldedness (gyrification).

## 2. Methods

In this study, we used publicly available neuroimaging resources and automated processing pipelines to analyze 595 brains from 12 species (Table 1). Among these species, surface area spans two orders of magnitude, with species distributed unevenly along that axis; therefore, to facilitate analysis, we divided them into four groups (small, medium, large, and x-large) based on natural breaks in the distribution of their surface areas. We first created or obtained reconstructed cortical surfaces for each species, including a template-based approach for the rhesus macaque and chimpanzee; an intensity-based approach for the bonobo and gorilla (Mangin et al., 1998); and acquiring preprocessed surfaces for humans (Cameron et al., 2013) and the rest of the species (Bryant et al., 2021; Ardesch et al., 2021a). With these surfaces, we then used our existing open-source computational pipeline (Demirci and Holland, 2022) to analyze patterns of brain morphology. All scripts generated for this study, including code sufficient to reproduce all figures, are available at https://github.com/mholla/NIMG23. Additionally, the data for all 595 subjects of all 12 species are available at doi.org/10.5281/zenodo.7574350, including the pial, white, and alpha surfaces, as well as cortical thickness, area, sulcal depth, curvature, and shape index at each vertex.

### 2.1. Preprocessing details

#### 2.1.1. Rhesus macaque

We obtained MR images of captive rhesus macaques from the publicly available PRIMatE Data Exchange (PRIME-DE) repository. These data come from multiple sites, which represents a challenge because different equipment and data acquisition protocols yield variations in data quality. Because of this, we specifically selected data acquired from the same brand of scanner (Siemens), with the same magnetic strength (3T) and pulse sequence (T1-weighted), and same subject scanning procedure (anesthetized). Additionally, we eliminated the sites with large surface errors from our analysis (Garcia-Saldivar et al., 2021) and restricted the age-span of subjects between 2.4 to 8 years. This resulted in the inclusion of 31 individuals (Table 1). Despite our selective use of only the most comparable data from the large PRIME-DE dataset, there are still slight differences in the data acquisition parameters (Table 2). Unregulated and non-harmonized data acquisition from multiple sites might lead to differences in cortical surface quality, which is especially important for comparative neuroanatomy studies (Hayashi et al., 2021). Data standardization and harmonization of public datasets are key to enable reproducibility of studies, so that multi-site bias can be reduced (Chen et al., 2014). Recently, several data acquisition and imaging protocols have been suggested to accelerate non-human primate neuroimaging progress (Milham et al., 2020). For example, (Autio et al., 2020) suggested using a specific type of receiver coil (24-ch radio-frequency (RF) receiving head coil) and following a specified image acquisition protocols for *in vivo* macaque imaging studies. Furthermore, a recent large exploratory study investigated the scanning-induced image variabilities and suggested a surface-based correction method for evaluating confounding effects (Chen et al., 2014). The study found that the variations in scanner and field strengths cause the most inconsistencies among the images, which we kept the same in this study.

We followed a template-based approach for processing the rhesus macaque scans, using the publicly available NMT (National Institute of Mental Health Macaque Template) template and accompanying single-subject bash scripts (Seidlitz et al., 2018). NMT is a high-resolution (0.25 mm isotropic) *in vivo* population-average template built from 31 rhesus macaques between the ages of 3.2 and 13.2 years, with three-class (white matter, gray matter, and CSF) tissue probability maps to show the probability of each voxel belonging to each specific tissue type.

**Fig. 2.**
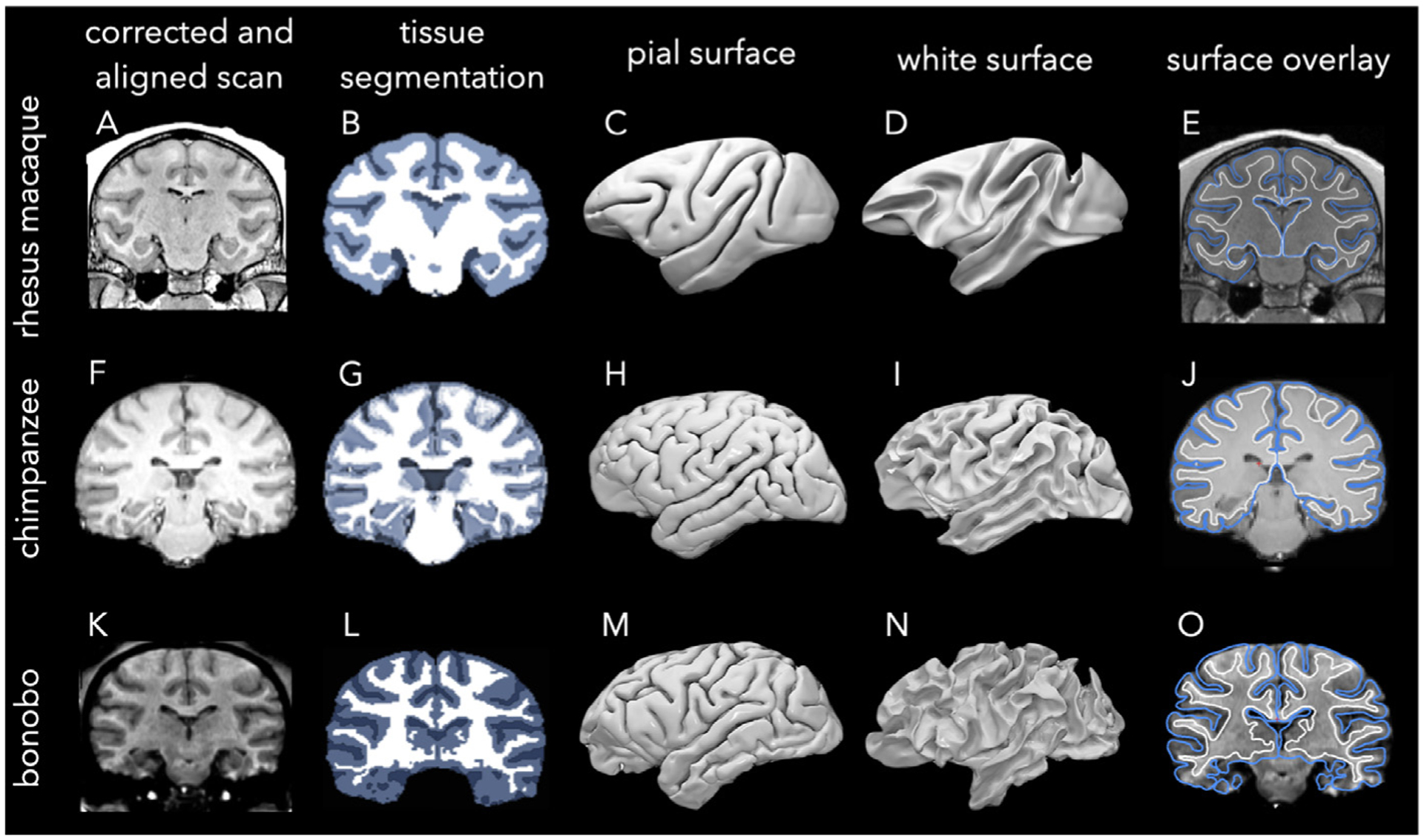
Preprocessing steps of the rhesus macaque, chimpanzee, and bonobo scans. Rhesus macaque scans from PRIME-DE were aligned to the NMT template and bias-field inhomogeneities were corrected (A) before 3-class tissue segmentation (B) and the reconstruction of pial (C) and white (D) surfaces using AFNI. Chimpanzee scans from NCBR were aligned to the Juna-Chimp template, bias-field corrected, and skull-stripped (F) before 3-class tissue segmentation (G) and reconstruction of pial (H) and white (I) surfaces using SPM and CAT. A bonobo scan from NCBR was bias-corrected, anatomically aligned, and skull-stripped (K) before 3-class tissue segmentation (L) and reconstruction of pial (M) and white (N) surfaces using BrainVisa Morphologist toolbox. The rightmost column shows the surface boundary estimations overlaid on top of MR slices for each species (E,J,O). Note that the segmentation of rhesus-macaque and chimpanzee subjects rely on the NMT and Juna-Chimp templates and species-specific segmentation methods, resulting in slight differences. For instance, the medial thalamus and lateral ventricles are within the inner compartment of the white matter surface in chimpanzees (J) but not in rhesus macaques (E) and the bonobo (O). However, this does not affect our cortical thickness estimations as thickness values that are less than 0.5 mm, including along the medial wall, are set to zero.

Using the bash scripts with slight changes in parameters and optional arguments where necessary, we ran the tools ANTs (Avants et al., 2011) and AFNI (Cox, 1996; Cox and Hyde, 1997) for processing individual rhesus macaque scans. Each individual scan was bias corrected, aligned, and registered to the NMT template both linearly and non-linearly (Fig. 2A). Using the NMT tissue prior masks, subject masks were generated in the template space, which were then transformed back to the native space for further analysis. ANTs was used for bias-field correction (*N4BiasFieldCorrection*), brain extraction (*antsBrainExtraction*), and tissue segmentation (*antsAtroposN4*) (Fig. 2B); and AFNI for image registration (*align_epi_anat.py*) and surface reconstruction (*IsoSurf*) (Fig. 2C–D).

AFNI’s *IsoSurf* uses the Lewiner’s marching cubes algorithm to create an isosurface from the input volume (Lewiner et al., 2003). After smoothing the initial surface using Laplacian and Taubin smoothing algorithms (Ohtake et al., 2001), topological defects were observed on pial and white surfaces. We used Freesurfer’s *mris_fix_topology* algorithm to automatically fix topological deformities such as gyral handles and sulcal holes. This algorithm ensures spherical topology of each cortical surface (pial and white), in which Euler’s number is 2. However, if the algorithm fails to fix all the defects, we intervened manually and corrected the segmentation volumes using ITK-SNAP http://www.itksnap.org/ (Yushkevich et al., 2006). More advanced topology correction algorithms might yield better outcomes for the estimation of the white surface, such as the HCP-NHP pipeline (Autio et al., 2020) or Topofit, which employs machine learning algorithms (Hoopes et al., 2022).

#### 2.1.2. Chimpanzee

We obtained 54 MR images of chimpanzee brains from the NCBR (supported by NIH grant NS092988) (Table 1). All the chimpanzees are from Yerkes National Primate Research Center (YNPRC) at Emory University Institutional Animal Care; 18 of them are mother-reared, 23 of them are nursery-reared, and the rest are wild (NCBR). All of the images were acquired at 3T and had been previously bias-corrected, denoised, and skull-stripped (Fig. 2F).

We followed a template-based approach for processing the chimpanzee brain scans, using the publicly available T1-weighted Juna-Chimp template and the accompanying structural processing pipeline and MATLAB SPM (Statistical Parametric Mapping) batch scripts (Vickery et al., 2020). This is a 1 mm-resolution *in vivo* population-average template built from 223 chimpanzee brains between the ages of 9 and 54 years, with three-class tissue probability maps (Vickery et al., 2020).

We used SPM12 (Ashburner, 2009), and the toolbox CAT12 (Computational Anatomy Toolbox) (Gaser and Dahnke, 2019), both run inside MatlabR2019b©, for processing individual chimpanzee scans. We registered each individual scan to the template by manually setting the stereotaxic origin at the anterior commisure to (0,0,0) x-y-z coordinates within SPM (Fig. 2F). Tissue segmentation (Fig. 2G) and white and pial surface reconstruction (Fig. 2H–I) were then performed using the same toolbox. We would like to note that the gyri on the top of the chimpanzee brain appears slightly thinner than other areas (temporal or frontal), likely due to a bias of MR signals (F, J).

#### 2.1.3. Bonobo and gorilla

We obtained MR images of one bonobo and one gorilla from the NCBR dataset, which were living in captivity. Both were T1 weighted images acquired at 1.5T (Table 1) (Rilling and Insel, 1999).

We followed an intensity-based approach for processing the bonobo and gorilla scans, using BrainVisa Morphologist toolbox (Cointepas et al., 2001) for skull-stripping, segmentation, and surface reconstruction. BrainVisa offers significant advantages as a fast automated processing pipeline with minimal manual interventions, an intuitive graphical user interface, and modular structure, and powerful and robust topological correction algorithms for surface reconstruction. Unfortunately, it is optimized for human brains (Rivière et al., 2009; Fischer et al., 2012) and only for T1-weighted images. Because of this, it is challenging to process non-human brains, especially the small brains, as manipulation of the original scan size distorts the spatial resolution. However, as bonobo and gorilla brains are relatively close in size to human brains, the software can be fine-tuned to account for their anatomical differences with humans.

The pipeline begins with the manual selection of four anatomical points. After inhomogeneity normalization (Mangin, 2000) (Fig. 2K), the tissue intensities are estimated (Mangin et al., 1998). Next, the hemispheres are split and the cerebellum and brain stem are removed. Finally, the gray and white matter are segmented (Fig. 2L) and the pial and white surfaces are reconstructed (Fig. 2M–N). As these images were acquired at 1.5T, they have slightly lower image quality, signal-to-noise ratio, and gray/white contrast. This results in small impurities in the segmentation, which is reflected on the reconstructed surface especially around sub-cortical regions. This might slightly impact our cortical thickness estimations in this region.

#### 2.1.4. Humans

We obtained preprocessed cortical surface reconstructions of 501 typical human brains from the publicly available multi-site neuroimaging data shared by the Autism Brain Imaging Data Exchange (ABIDE-I) repository (Di Martino et al., 2014; Cameron et al., 2013). While 573 subjects are available in the database, we excluded 72 subjects based on scan quality (Pardoe et al., 2016), as in our previous work (Demirci and Holland, 2022). MRI data acquisition parameters and scanner types varied between sites, but all scans were acquired at 3T. Detailed information regarding the functional and anatomical scan parameters of each site can be found in the supplementary information of Di Martino et al. (2014).

#### 2.1.5. Remaining species

Each of the remaining seven non-human primate species were originally obtained from the Netherlands Institute of Neuroscience Primate Brain Bank (PBB; http://www.primatebrainbank.org/); all of the animals were living in captivity in Dutch zoos and primate centers. We obtained the preprocessed cortical surface reconstructions of each specimen from Bryant et al. (2021), who produced these surfaces using Freesurfer, FSL, ANTs, and MATLAB with manual corrections where necessary (Ardesch et al., 2021b). For the tissue segmentation of the smallest brain samples, a three-step registration was used by Bryant et al. (2021), first registering the image to a macaque template, then to a chimpanzee template, and lastly to the human Talairach space, before warping back to the initial native space (Ardesch et al., 2021b).

### 2.2. Processing pipeline

**Fig. 3.**
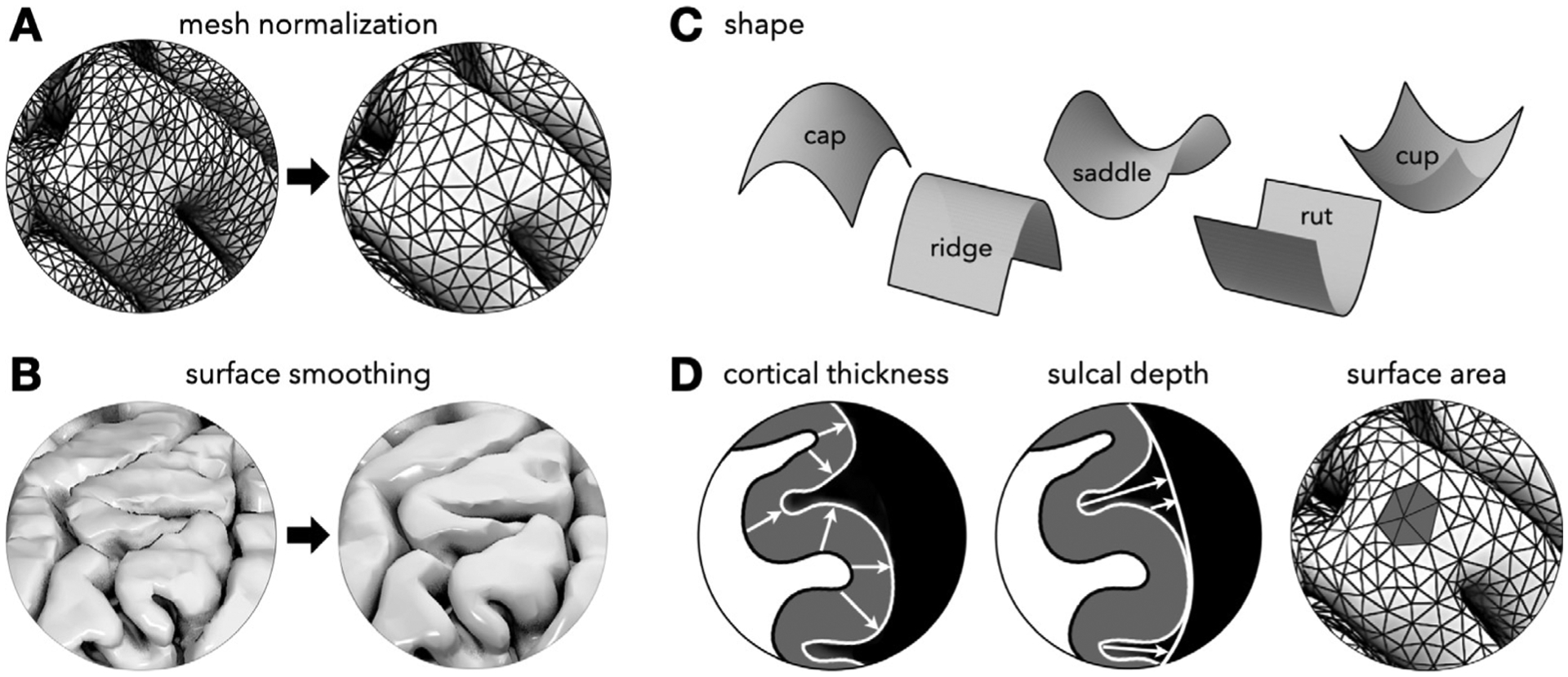
Our open-source, surface-based, brain morphology pipeline (https://github.com/mholla/curveball). The pipeline works with triangulated pial and white surfaces. First, the surface mesh is normalized (A) and smoothed (B). Then the local shape is extracted by calculating curvature and shape index at each point (C). Additionally, cortical thickness, sulcal depth, and surface area are measured (D). Full details of the pipeline can be found in Demirci and Holland (2022).

From the triangulated surface meshes produced by the workflows above, we used our open-source computational pipeline (Demirci and Holland, 2022) (Fig. 3) to calculate cortical thickness, curvature (Gaussian and mean), shape (shape index), sulcal depth, and surface area at each point of the pial surface for both hemispheres. The total number of points varies for each species (see Supp. Table 1). The vertex densities for each species are carefully determined in order to prevent over/undersampling. We calculated the vertex density for each species by dividing the total surface area by the total number of vertices and taking the square root. Then, The resolutions are ~ 2 mm, ~ 1.5 mm, ~ 1 mm, and ~ 0.5 mm for x-large, large, medium, and small species, respectively (for full data see Supp. Table 1). The resolution increases as size gets smaller, in order not to under-sample the smaller sized species. Based on this analysis, our species-specific meshes approximate the spacing and resolution of the corresponding MR images.

The full details of the pipeline are explained elsewhere (Demirci and Holland, 2022), but in brief, it starts by normalizing (Fig. 3A) and smoothing (Fig. 3B) the mesh to increase robustness, reduce single vertex errors, and obtain mesh elements of approximately equal size. Both Laplacian and Taubin smoothing algorithms are applied only to an extent, in which over-smoothing and shrinkage of the surface is avoided (Demirci and Holland, 2022). We employed both of the surface denoising algorithms because, in our experience, Taubin smoothing itself is not sufficient to remove the single-vertex errors for robust and accurate morphometric calculations. We also used Laplacian smoothing with caution as it can change the shape of the surface drastically by shrinking it, especially at higher iterations; Taubin smoothing, on the other hand, preserves the overall geometry of the surface by first shrinking the surface, and then inflating it back with a greater magnitude compared to the initial scaling parameter. We also ensured that the normalized and smoothed pial and white surfaces have very similar total number of vertices by utilizing mesh decimation and/or subdivision with a preset target number of triangles.

Then, using the principles of discrete geometry, intrinsic and extrinsic curvatures (Gaussian and mean curvature, respectively) are calculated. These curvatures are then used to determine the local shape via the shape index (Koenderink and van Doorn, 1992), which is a non-dimensional measure that characterizes the local shape as a single scalar between −1 and 1. This corresponds to a perfect cup and cap, respectively, with additional shapes in between (Fig. 3C). Shape index has been used previously to analyze the structure of the cortex (Hu et al., 2013; Shimony et al., 2016; Kim et al., 2016), although not as frequently as other surface measures, such as Gaussian, mean, and principal curvatures. We also note that estimation of the shape index is highly dependent on the spatial resolution of the data acquisition and the accuracy of the cortical reconstruction as true with any other measure, which is why normalization and smoothing of the surface are critical.

Next, we calculate the local cortical thickness and sulcal depth as linear Euclidean distances between the closest points on different surfaces. Cortical thickness is the average distance between points on pial and white surfaces (that is, the average of the distance from a point on the white surface to the closest point on the pial surface, and the distance from that point back to the nearest point on the white surface). Sulcal depth is the distance between points on the pial surface and an alpha surface that tightly wraps the cortex (Demirci and Holland, 2022) (Fig. 3D). Alpha values to generate alpha surfaces for each species varies and the values are correlated with size of the species (see Supp. Table 1). The alpha values were chosen manually as the minimum alpha value that tightly wraps the whole cortical surface. All local surface measures are smoothed by applying two iterations of weighted-averaging, and then they are averaged to yield the average cortical thickness and the folding amplitude (average sulcal depth). The pial and alpha surface areas are also calculated, as the sum of each triangular element area on the respective surface. From these, the dimensionless gyrification index (GI) can be found; GI quantifies the degree of foldedness as the ratio between the total and exposed surface areas (pial and alpha, respectively) (Zilles et al., 1989). We also calculated the total cerebral volume for each species using our pipeline. Finally, we calculate the cortical thickness ratio, defined as the ratio between the average thickness of all convex points to all concave points.

## 3. Results

### 3.1. Validation of calculations

**Fig. 4.**
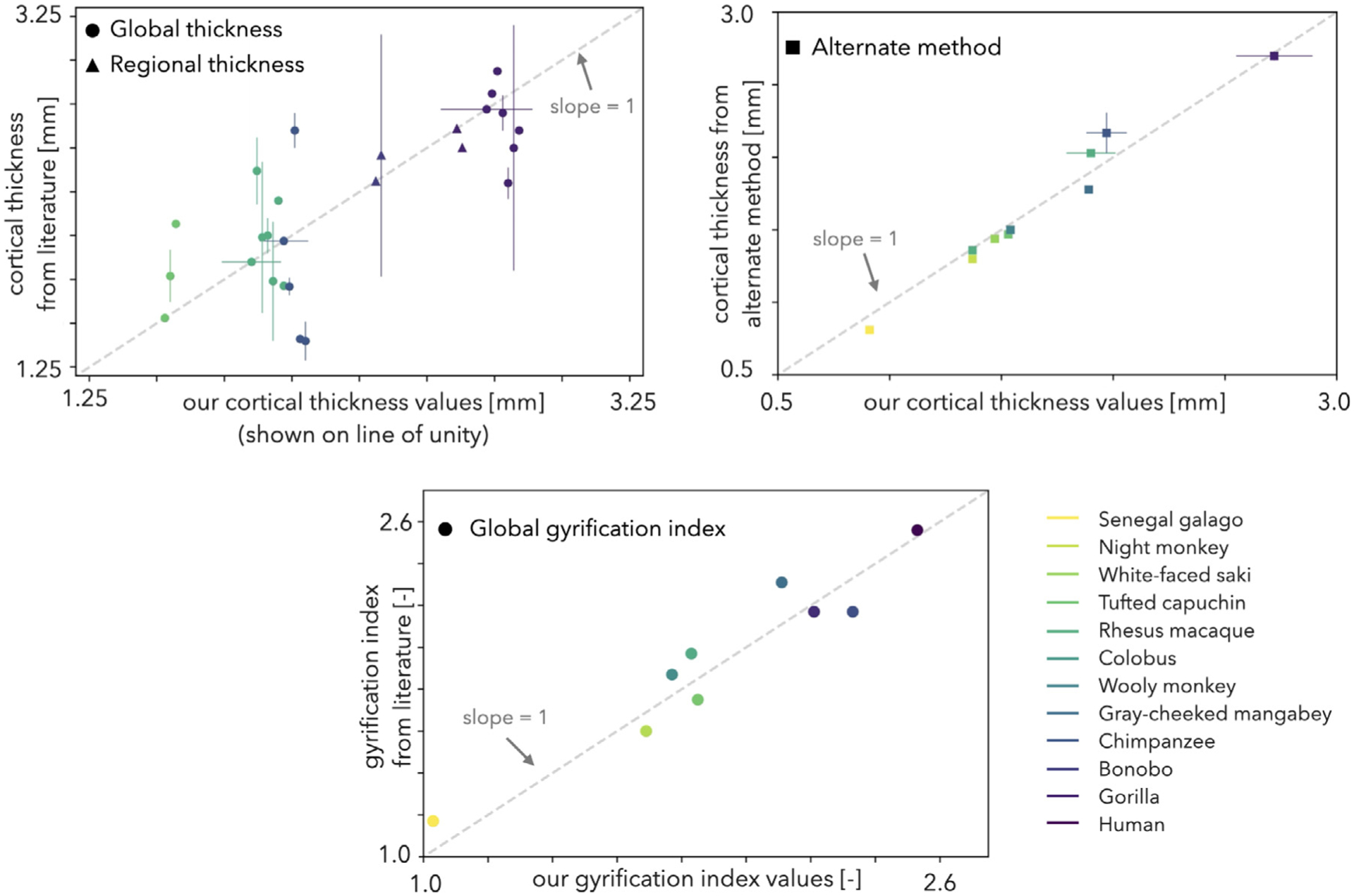
Comparison of our global cortical thickness (top) and gyrification index (GI, bottom) values with data collected from literature for the 12 species investigated. The dashed lines represent unity. Global data (●) were collected when available; if not, regional data (▲) were averaged to get a global estimate. Error bars represent the cumulative standard deviation of all standard deviations collected from each publication. A methodological comparison was also conducted comparing results for the same specimen from our computational pipeline to Freesurfer’s and CAT12’s surface-based morphometry approach (humans and chimpanzees respectively) and ANT’s volumetric cortical thickness algorithm (rhesus macaques) (■). Sources for cortical thickness data are listed in Table 3; all GI values were found in Zilles et al. (2013). The full cortical thickness data represented in this figure is available on Github (https://github.com/mholla/NIMG23).

To validate our calculations, we collected cortical thickness and GI values from the literature (Table 3) and compared them to our own (Fig. 4). In some cases, global values were found in the literature and a comparison was straightforward. However, these values were not available for some species, especially those that are not common research models (Fig. 4, top left). In the event that we could not find global data for a species, we first looked for regional data and, if found, took its average to obtain an estimate of the global value (Fig. 4, top left).

Additionally, we compared our results to those obtained from a different method on the same surfaces (Fig. 4, top right). We include these comparisons, in which the same subject is compared to itself via different methods, as an evaluation of our computational method. For example, we compared our cortical thickness findings for seven species from Bryant et al. (2021) to their results from Freesurfer. Moreover, we compared our cortical thickness data of rhesus macaque subjects against volumetric cortical thickness data obtained by ANTs’s *KellyKapowski* algorithm, which is outputted by the pipeline provided by Seidlitz et al. (2018). We also compared our chimpanzee results against surface-based cortical thickness data obtained through CAT12. The slight differences between the results (Fig. 4, top right) might be due to the additional smoothing and normalization by our pipeline (Clarkson et al., 2011), which is indispensable for robust and accurate curvature measurements.

The strong alignment of our values with previously reported data supports our computational pipeline and calculations. However, the alignment of prior global thickness with our calculations does not speak directly to the accuracy of our thickness comparisons between gyri and sulci within each species. Unfortunately, as this is the first study to investigate gyral and sulcal thickness differences in non-human primates, this type of validation is not possible due to lack of data.

### 3.2. Relationships between cortical thickness and shape

**Fig. 5.**
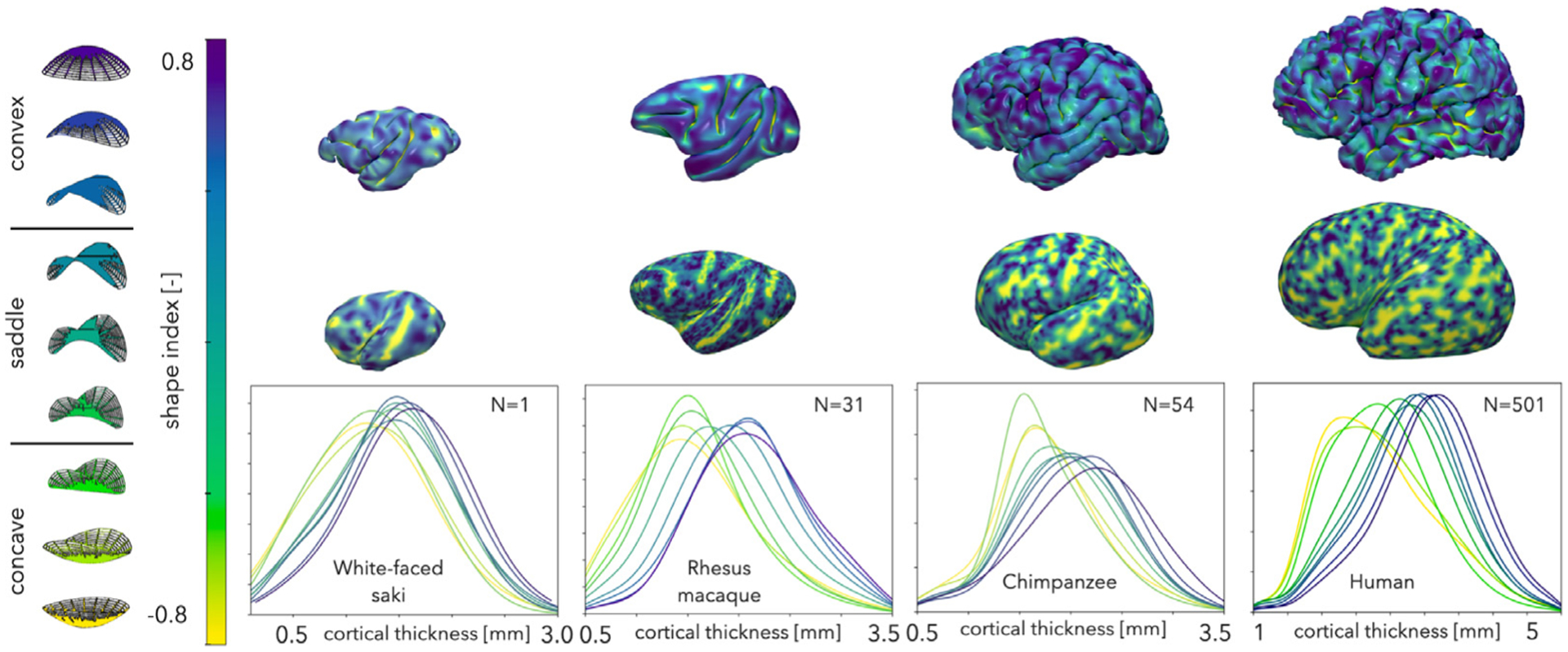
Correlation of cortical thickness with cortical geometry. Top: shape index is overlaid onto the pial surface of a representative small (white-faced saki), medium (rhesus macaque), large (chimpanzee), and x-large (human) brain. Middle: Inflated pial surfaces with shape index overlaid. Bottom: Cortical thickness kernel density distribution profiles with respect to local shape, aggregated for *N* = 1 white faced saki, *N* = 31 rhesus macaques, *N* = 54 chimpanzees, and *N* = 501 humans, are shown. Cortical thickness decreases consistently from convex to saddle to concave shape.

To understand the relationship between cortical thickness and shape, we next investigated the cortical thickness distribution for points of a given shape. Here we consider the nine distinguishable shapes distinguished by the scale-invariant shape index (Koenderink and van Doorn, 1992): cup, trough, rut, saddle rut, saddle, saddle ridge, ridge, dome, and cap, with shape index values ranging from −1 to 1. When we considered the distribution of shape index in each species, we noticed that increases in size and the degree of foldedness lead to sulcal invaginations and an increase in the frequency of concave points (Supp. Fig. 1). After separating all points with respect to their shapes using the shape index, we extracted the cortical thickness of all points corresponding to each particular shape, aggregated them among all subjects, and plotted them as a kernel density estimation (Fig. 5). Here, we depict only one species from each group (small, medium, large, and x-large), but these results are similar for all of the species we investigated (Supp. Fig. 2). In each case, cortical thickness has a unimodal distribution and varies along a gyral–sulcal spectrum from the convex cap shape, which is the thickest, to the concave cup shape, which is the thinnest. Additionally, we demonstrated the same trend in each subset of the ABIDE dataset for humans, confirming the site independence (Supp. Fig. 3). The non-human primates show very similar trends to those previously seen in human brains (Demirci and Holland, 2022), although the difference between cup and cap shapes seems to be smaller for smaller and less folded brains.

**Fig. 6.**
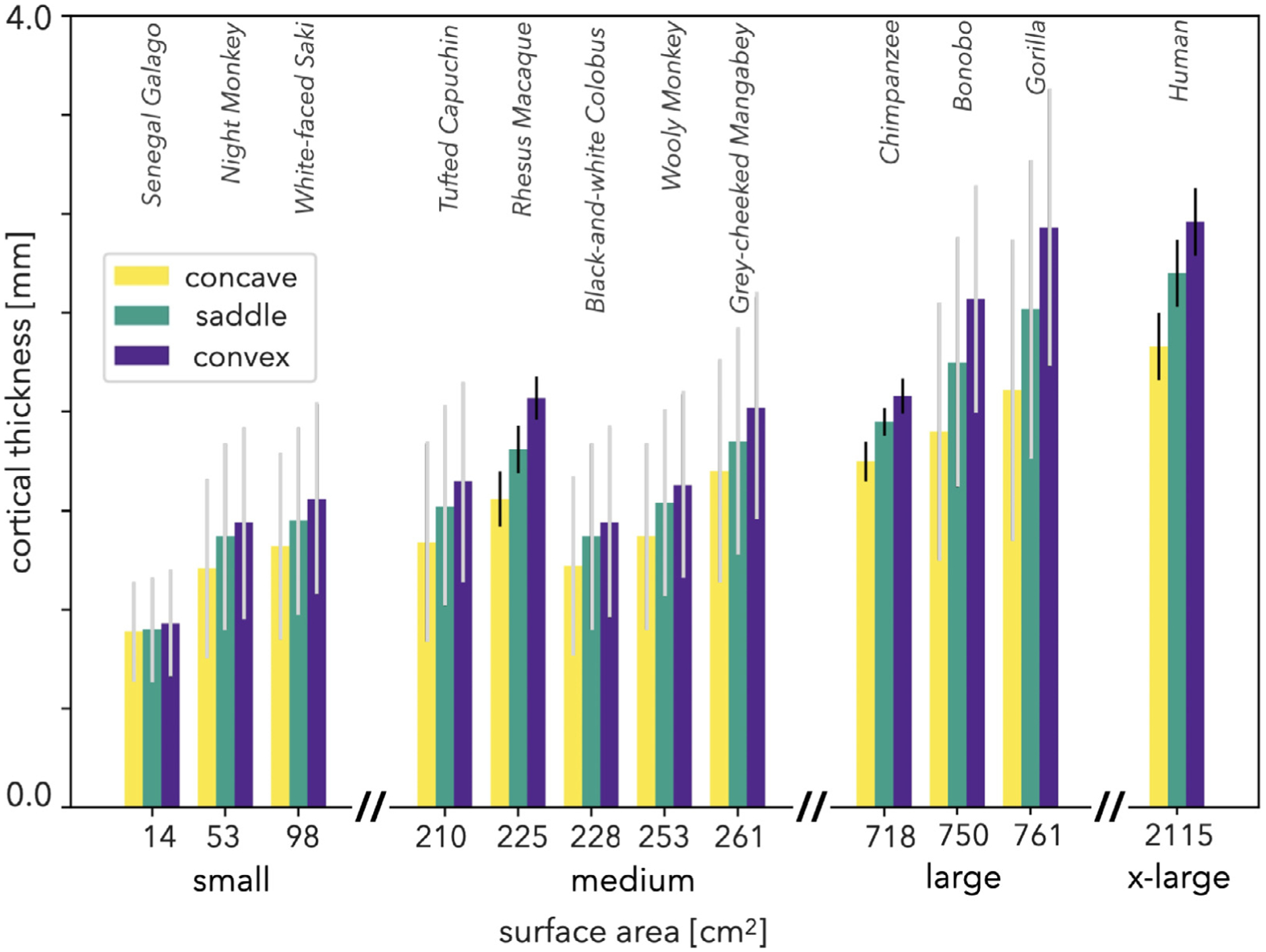
Average cortical thickness of all concave, saddle, and convex shaped points in 12 primate species with respect to total cortical surface area of each species. Cortical thickness is greatest for convex shapes, smallest for concave shapes, and in the middle for saddle shapes for all species. *N* = 31, *N* = 54, and *N* = 501 for rhesus macaques, chimpanzees, and humans respectively. Error bars represent one standard deviation. For species with single subjects, gray error bars represent variation within subjects. For species with multiple subjects, black error bars represent variation across subjects.

To investigate similar trends in all 12 species, we considered a more simplified set of shapes, consisting of convex (cap, dome, and ridge, with −1 < SI < −0.375), saddle (saddle ridge, saddle, and saddle rut, with −0.375 < SI < 0.375), and concave (rut, trough, cup, with 0.375 < SI <1) shapes. After separating all points with respect to these three shapes using the shape index, we extracted the cortical thickness of all points corresponding to each particular shape, aggregated them among all subjects, and plotted the average thickness (Fig. 6). When multiple subjects were available, we also calculated the standard deviation of subject averages, while for single subjects we calculated the variation within the subject.

Across the range of species, we found three general trends. First of all, cortical thickness tends to increase as surface area increases, which has long been known (Hofman, 1985). This is not strictly always true; in particular the rhesus macaque appears to be unexpectedly thick. However, note that the species are not equally distributed along the range of surface areas; the differences *within* groups (< 100 cm^2^) are much smaller than the differences *between* groups (> 100 cm^2^). Secondly, for every species, the average cortical thickness always increases from concave to saddle to convex shapes (Fig. 6). Finally, there appears to be a general trend for the thickness difference between concave and saddle shapes, and between saddle and convex shapes, to increase along with increases in surface area. For instance, in the Senegal galago the thicknesses are barely different (*p* = 0.01, *d* = 0.04 and *p* < 10^−3^, *d* = 0.11 for concave-saddle and convex-saddle comparison, respectively), while in the great apes (large and x-large groups) the differences are much more significant (*p* = 10^−10^, *d* ≈ 0.60 for both concave-saddle and convex-saddle comparison).

**Fig. 7.**
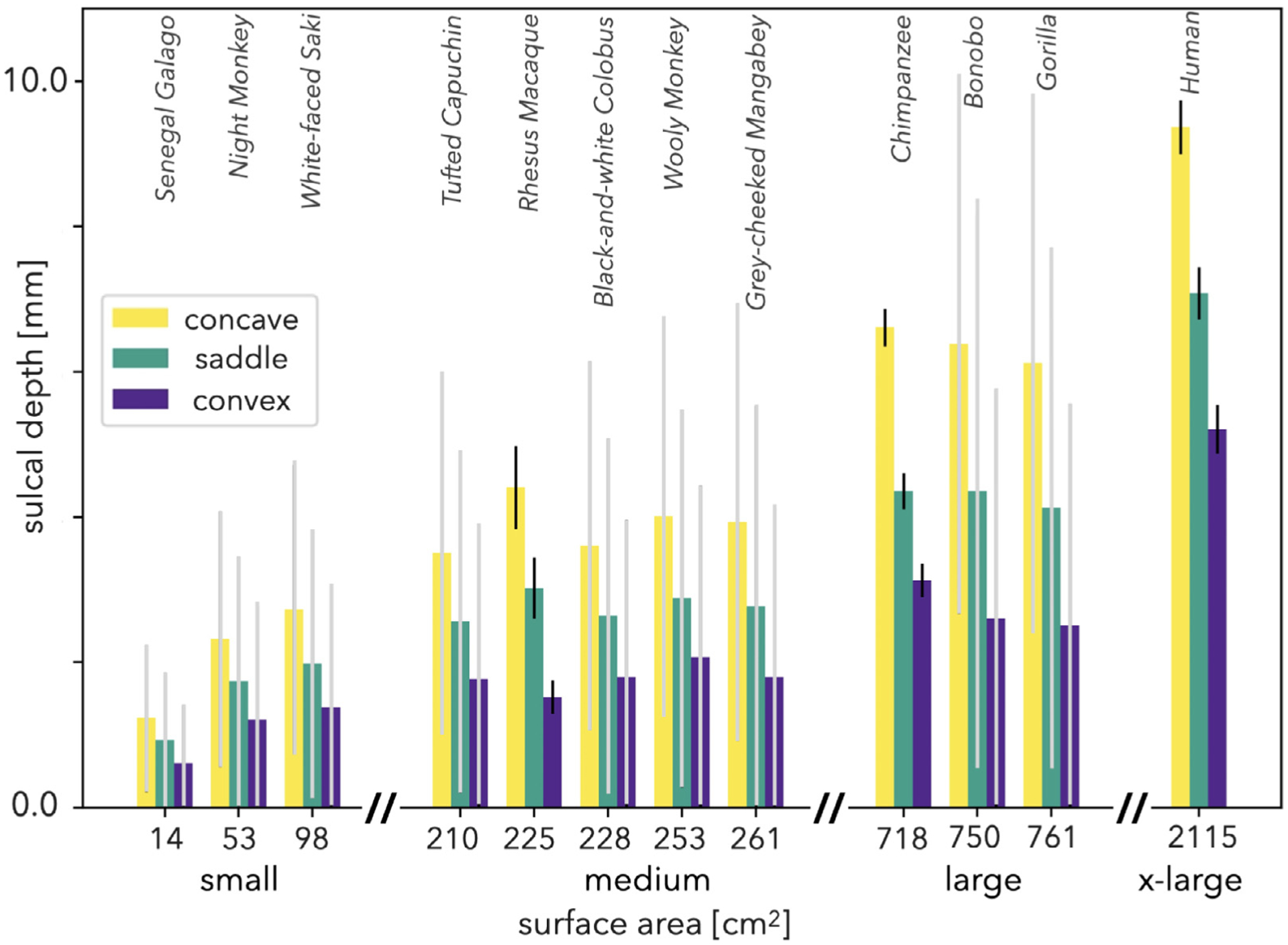
Average sulcal depth of all concave, saddle, and convex shaped points of the cortex with respect to total cortical surface area of each species. Convex points are clustered on gyral ridges, concave points on sulcal valleys, and saddle points in the middle. *N* = 31, *N* = 54, and *N* = 501 for rhesus macaques, chimpanzees, and humans respectively. Error bars represent one standard deviation. For species with single subjects, gray error bars represent variation within subjects. For species with multiple subjects, black error bars represent variation across subjects.

We also remark that the location of these shapes throughout the cortex are highly consistent (Fig. 7). Convex points are more superficially located, concave points reside deeper, and saddle points are located mostly in between for each species. In addition, the average distance between gyri and sulci increases in tandem with size. These analyses shows that, there is a strong relationship between cortical thickness, folding amplitude, and shape of the cortex globally. Another important remark is the differences in variation of cortical thickness and folding amplitude within and across subjects of the same species. As expected, local variations of the measures within a single subject are much higher than the variations of average measurements across subjects of the same population. Average cortical thickness and folding amplitude corresponding to three shapes vary less compared to the variation across subjects of a single population. As an example, the standard deviation of local cortical thickness for a single chimpanzee subject (or all local values for the whole population) yields ~ 0.5 mm, however the standard deviation of average cortical thickness across all subjects within the chimpanzee population is only ~ 0.1 mm.

### 3.3. Intraspecies variations in brain morphology

**Fig. 8.**
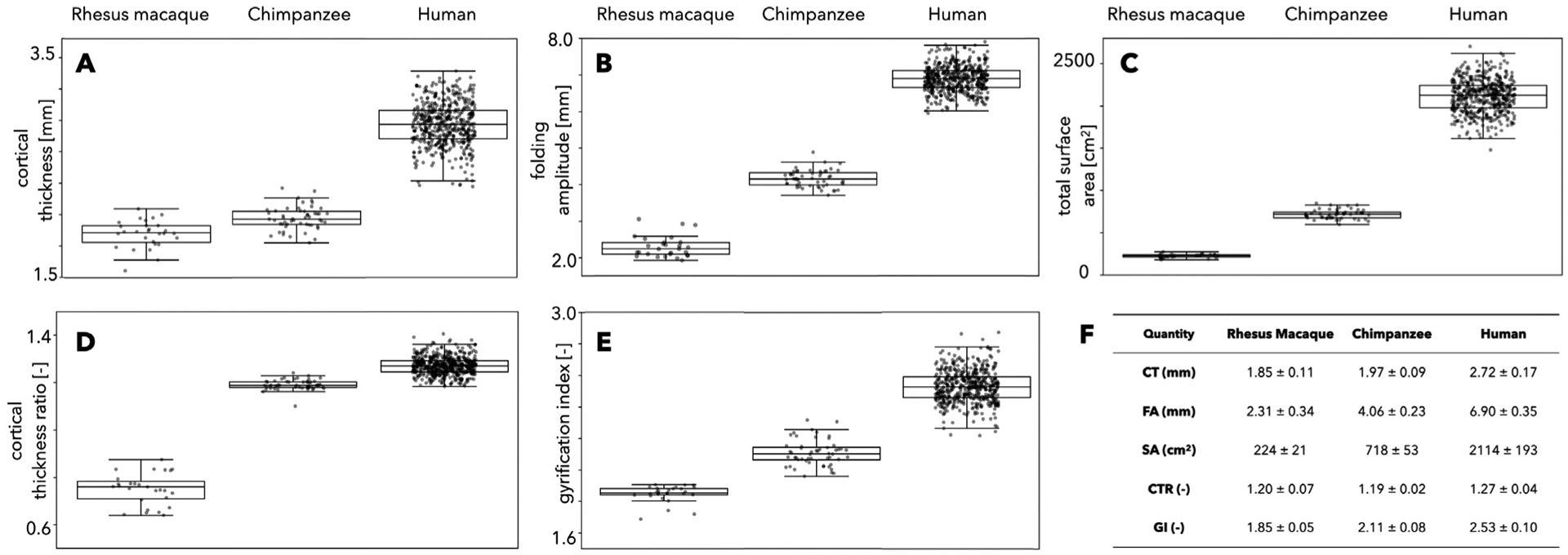
Intraspecies variability of A) average cortical thickness, B) folding amplitude, C) total surface area, D) cortical thickness ratio, and E) gyrification index, for N=31 rhesus macaques, N=54 chimpanzees, and N=501 humans. Average values (± standard deviation) for all subjects from each species are listed in F.

To understand the extent of variations between individuals, we analyzed intraspecies differences in average cortical thickness (Fig. 8A), folding amplitude (Fig. 8B), total surface area (Fig. 8C), cortical thickness ratio (Fig. 8D), and GI (Fig. 8E). Average values (± standard deviation) for all subjects from each species are also shown (Fig. 8F). This was possible only for the three species for which we had multiple subjects: N=31 macaques, N=54 chimpanzees, and N=501 humans. Overall, we observed that humans appear to have the largest variation between subjects, particularly in the average cortical thickness; this could be a result of the significant age range in the subject pool, from 7 to 56 years, during which cortical thickness evolves significantly.

### 3.4. Interspecies variations in brain morphology

**Fig. 9.**
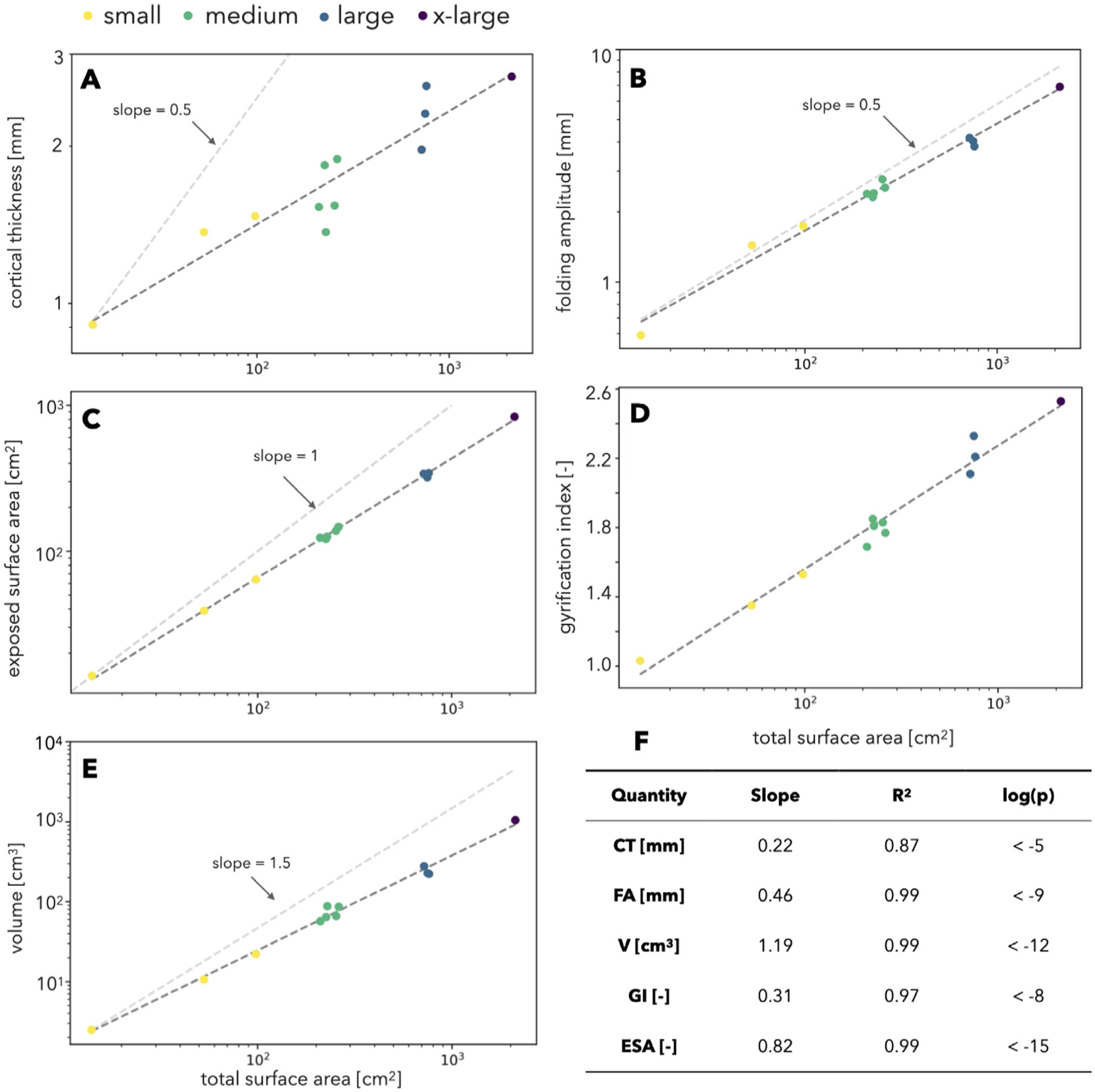
Allometric scaling relationships of measures of brain morphology with surface area. Data from 12 primate species are shown for cortical thickness (A), folding amplitude (B), exposed surface area (C), gyrification index (D), and volume (E). A power regression was performed, and the line of best fit is shown, with the slope and correlation values listed (F). Additionally, for dimensioned quantities, the predicted relationship in the case of isometric scaling is indicated with gray dotted lines for comparison.

In order to better understand changes in brain morphology across primate brains of different forms and sizes, we determined the allometric scaling of several anatomical parameters against total surface area, including average cortical thickness, folding amplitude, brain volume, GI, and exposed (i.e. alpha) surface area (Fig. 9). We considered log–log relationships for the dimensioned quantities (cortical thickness, folding amplitude, exposed surface area, and brain volume) and semilog relationships for the dimensionless quantity of gyrification index. Additionally, for the dimensioned quantities, we compared their scaling against the expected relationship in the case of isometric scaling or geometric similarity (dashed, light-gray lines in Fig. 9), in which lengths would scale with the 1/2 power of total surface area, surface area would scale linearly (first power), and total brain volume should scale with 3/2 power (Im et al., 2008).

All the investigated quantities correlate significantly with total surface area, increasing as the surface area increases. However, the amount of increase differs by several orders of magnitude: surface area increases by more than 100 fold between the smallest and largest species, but cortical thickness increases only by threefold (Fig. 9A) and folding amplitude by sevenfold (Fig. 9B). In comparisons with isometric scaling relationships, cortical thickness, volume, and exposed surface area scale slower than expected, while folding amplitude is surprisingly very similar to isometric scaling. Additionally, volume, gyrification index, folding amplitude, and exposed surface area are very strongly correlated with total surface area (*R*^2^ ≥ 0.97), while cortical thickness does not have as strong a correlation (*R*^2^ = 0.87). This might represent an indirect interaction between the two.

**Fig. 10.**
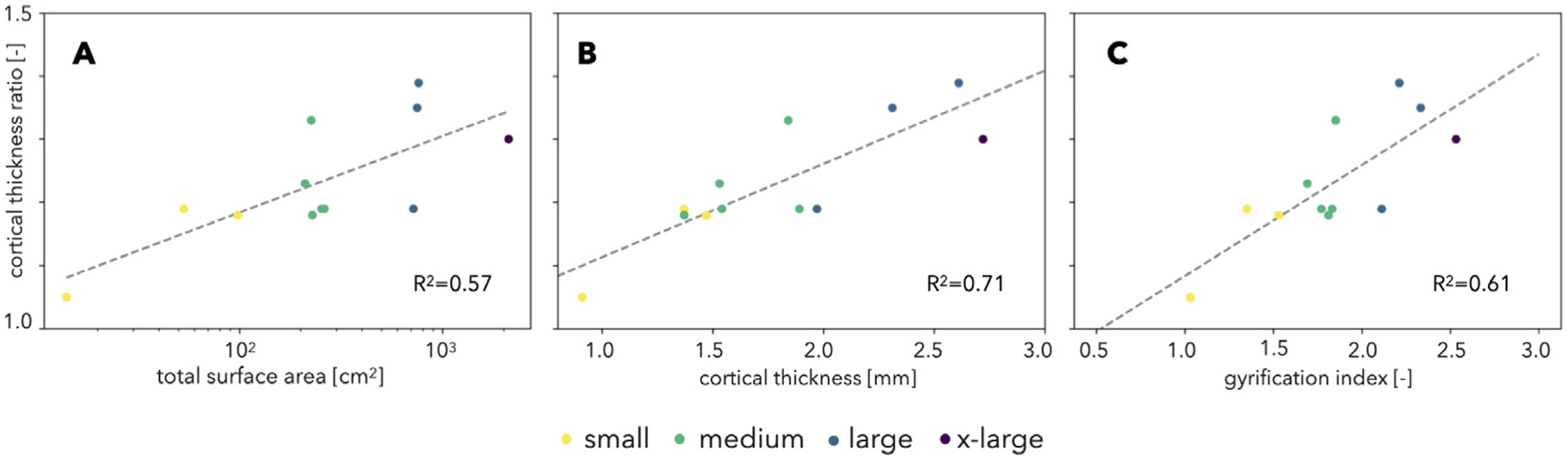
Cortical thickness ratio correlates significantly with increase in total surface area (left), average cortical thickness (middle), and foldedness (GI) (right).

We also investigated the correlation of convex–concave cortical thickness ratio with other parameters of brain morphology (Fig. 10). Cortical thickness ratio was observed to increase with surface area, cortical thickness, and foldedness (GI). Similar to cortical thickness, cortical thickness ratio also has an indirect relation to the increase in surface area.

### 3.5. Atrophy of cortex with age

Atrophy of the cortex as a function of age is well-known for humans (Salat et al., 2004; Buckner et al., 2004; Hurtz et al., 2014; Fjell et al., 2015; Amlien et al., 2016). Here, we investigated the atrophy of cortical thickness with aging for both chimpanzees and humans. Additionally, we analyzed the variation of cortical thickness ratio for both chimpanzees and humans with respect to aging to observe any significant correlations. Aging analysis was not possible for rhesus macaques as age information of subjects are not publicly available for all sites.

**Fig. 11.**
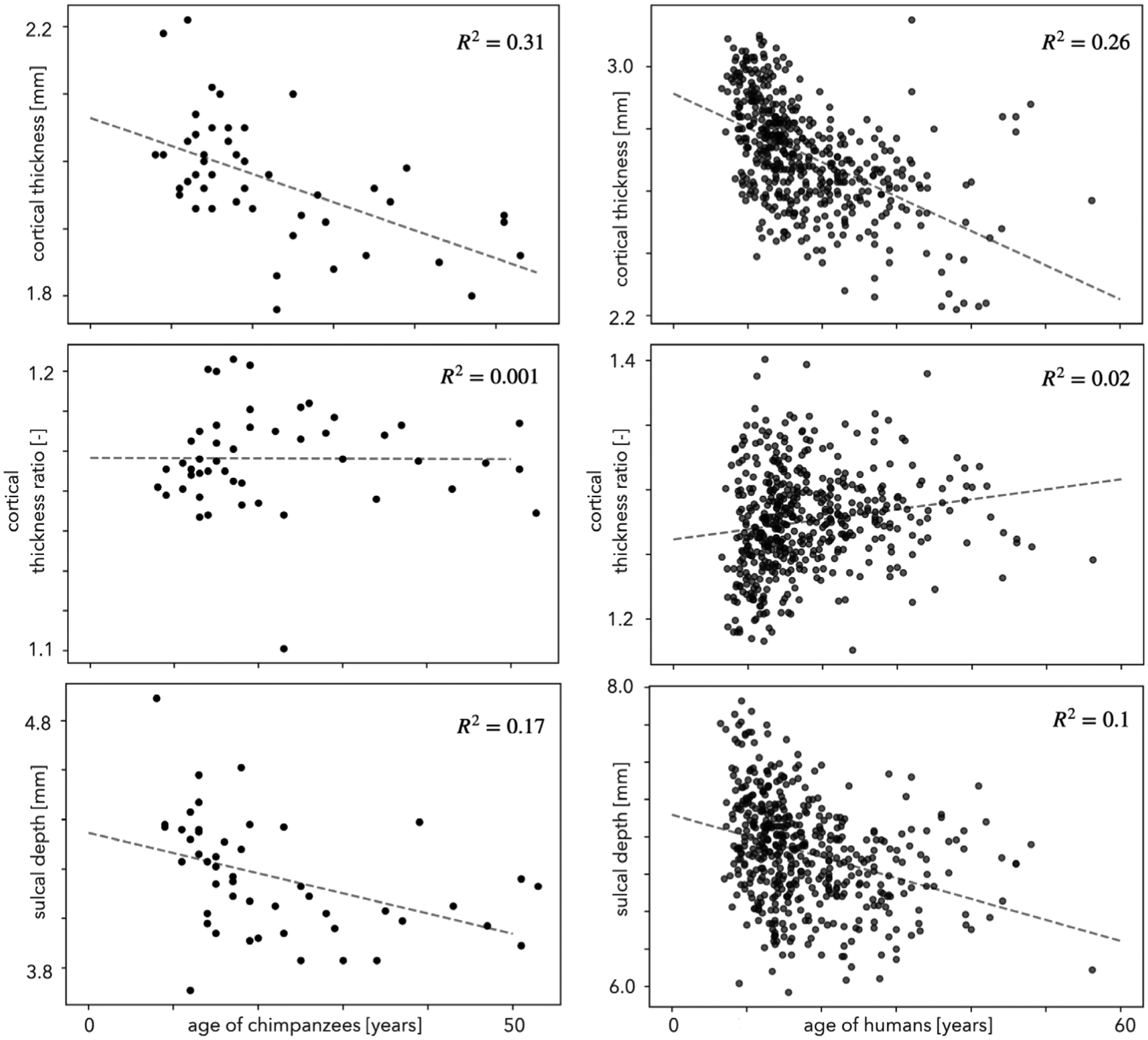
Changes in cortical thickness, cortical thickness ratio, and sulcal depth with respect to aging for humans and chimpanzees. Top: Atrophy of the cortex with aging for both species. Middle: Cortical thickness ratio does not change considerably with age. Bottom: Cortical atrophy causes sulcal shallowing for both species.

Our results demonstrate cortical thinning in both species (*p* ≪ 10^−10^ for humans and *p <* 10^−5^ for chimpanzees, Fig. 11, top) similar to earlier studies (Amlien et al., 2016; Vickery et al., 2020) . Therefore, chimpanzees – humans’ closest relative – exhibit age-related cortical atrophy similar to humans that might be due to similar evolutionary patterns (Vickery et al., 2020). However, the thickness ratio does not seem to change systematically with age (Fig. 11, middle). This suggests that the cortical thickness of gyri and sulci both decrease proportionately during aging for these species. We also plotted the average sulcal depth across age and found a significant decrease (Fig. 11, bottom), (*p* < 10^−13^ for humans and *p* < 0.005 for chimpanzees), suggesting that cortical atrophy causes a decrease in depth (Yun et al., 2013).

## 4. Discussion

### 4.1. Primate brains change form as they increase in size

If all brains were geometrically similar (with size changing isometrically between them without changes in form), then all brains would be smooth like those of tarsiers and lemurs, and humans would have a thicker cortex (Fig. 9A). Instead, brains change in form as they change in size — this is true both for individual humans (Im et al., 2008), and across different species (Hayashi et al., 2021; Hofman, 2014). Larger brains tend to be gyrencephalic or folded (Mota and Herculano-Houzel, 2015; Zilles et al., 2013; Hofman, 1985; Rogers et al., 2010; Tallinen et al., 2014), and the larger they are, the more folded they tend to be — although interestingly, there are a few notable exceptions to this, such as the manatee, koala, and beaver, that require further investigation (Welker, 1990; Toro and Burnod, 2005). Conversely, cortical thickness varies much less than would be expected in isometric scaling (Hofman, 1985). Our results in 12 primate species closely follow these previous studies (Zilles et al., 2013; Hofman, 1985, 1988b; Rilling and Insel, 1999; Mota and Herculano-Houzel, 2015; Herculano-Houzel et al., 2008), showing increases in cortical thickness, folding amplitude, gyrification index, and brain volume with increasing surface area, without the scaling needed to maintain geometric similarity (Fig. 9). Similar observations portray the increase in degree of foldedness during human (Armstrong et al., 1995) and monkey ontogeny (Sawada et al., 2012).

### 4.2. Primates experience cortical atrophy and sulcal shallowing due to aging

We demonstrated significant cortical atrophy and sulcal shallowing for both chimpanzees (aged between 8–53) and humans (aged between 7–56) across age from childhood to late adulthood. Among the chimpanzees, 40% are older than 19 years old and 15% are younger than 11 years old. Among our human subjects, 25% are older than 22 years old and 36% are younger than 13 years of age. Therefore the chimpanzees represent an elderly population compared to the humans. (While the two neurodevelopmental timelines do not correspond exactly, they are close; a 13 year old human translates to a 11 year old chimpanzee, and a 22 year old human translates to a 19 year old chimpanzee (Charvet, 2021).) Despite these differences, global thinning of the cortex is common for both species (Fig. 11, top), similar to findings of Vickery et al. (2020) and contrary to Autrey et al. (2014) and Sherwood et al. (2011), in which they found no change in thickness. Our detailed analysis results suggest cortical thinning of chimpanzee brain at later stages of life, however significant thinning is present for both younger and elderly human populations starting at age 7. Local investigation of cortical atrophy reveals a proportional decline of cortical thickness at both gyri and sulci, such that cortical thickness ratio does not change much with age (Fig. 11, middle). In addition to atrophy of the cortex, we observed significant decrease of average sulcal depth (folding amplitude) for both species (Fig. 11, bottom).

### 4.3. Non-isometric scaling of surface area with volume leads to gyrification

Exposed surface area is observed to grow more slowly than total surface area (Fig. 9E), indicating an increase in folding. Exposed surface area increases significantly slower than predicted by isometric scaling; the 0.82 power between exposed and total surface area corresponds to a 1/0.82 = 1.22 power between total and exposed surface area, which is very close to the 1.25 reported previously (Hofman, 1988a) This can also be seen in the gyrification index, which would be one for a completely smooth brain. Instead, it is greater than one for all primate species (Fig. 9D), and increases with surface area, as found before (Pillay and Manger, 2007; Zilles et al., 1989; Tallinen et al., 2014; Essen et al., 2019).

Similarly, brain volume scales significantly slower than predicted by geometric similarity (Fig. 9C) (Hofman, 2012; Toro and Burnod, 2005); the 1.16 power between volume and surface area corresponds to a 1/1.16 = 0.86 power between surface area and volume, which is very close to the 0.9 reported previously (Hofman, 1988a).

The strength of the power law relationships suggests that geometrical quantities might depend on the overall size of the cortex, which could be the driving factor of gyrification (Herculano-Houzel, 2015; Essen et al., 2019).

### 4.4. Folding amplitude scales isometrically, but cortical thickness does not

Folding amplitude increases with surface area (Fig. 9B), as found before (Hopkins et al., 2014), and its scaling is not significantly different than the predicted isometric relationship (Fig. 9B). This has also been shown in a study of only humans (Im et al., 2008), although it is important to note that the rules that govern scaling within a species might be different than those that govern relationships between species. Interestingly, we demonstrated the isometric scaling of folding amplitude contrary to stable folding wavelengths among different sized brains (Heuer et al., 2019).

We note that our measures of folding amplitude are lower than the previously published folding depth values from (Heuer et al., 2019). We believe this difference is due to the different methods utilized. Heuer et al. (2019) used a global approximation to calculate the folding depth, by measuring the sulcal volume from a convex hull, divided by folding length. On the other hand, we employed a more local approach, calculating the sulcal depth of each point on the cerebral surface, evaluated relative to the alpha surface, and averaging all the values. In our experience, the alpha surface method yields a more accurate estimation of the depth than the convex hull, as it wraps the cerebral surface more tightly, outlining the concave medial temporal lobe and inferior medial regions without extending into the sulcal valleys. A convex hull, on the other hand, is similar to an alpha surface with higher alpha parameter; it does not fully outline the major curves of the cortex (see Fig. 5 in Demirci and Holland, 2022). Therefore, a convex hull might yield higher values of depth as it fails to capture the complex form of the brain.

Cortical thickness also increases significantly with increases in surface area, but its slope is significantly less than predicted (Fig. 9A) (Hofman, 1988b). This result supports previous findings that, cortical thickness correlates negatively with the degree of folding (Mota and Herculano-Houzel, 2015; Welker, 1990; Zilles et al., 2013; Hofman, 1985; Zilles et al., 1989; Toro and Burnod, 2005). For example, it has been shown in chimpanzees that increased foldedness (deeper sulci) in one hemisphere correlates with increased surface area and decreased cortical thickness (Hopkins et al., 2015); increased white matter surface area in one hemisphere is similarly associated with lower cortical thickness (Hopkins and Avants, 2013). Together, this suggests that an increase in total surface area leads to greater folding amplitude, but also to decreased cortical thickness in order to balance the total amount of gray matter occupied in constrained cranial volume for individuals within species.

Another factor could be that thinner cortices are easier to fold; this is true both regionally and globally. In humans, thinner regions have been found to be more folded than thicker regions (Van Essen et al., 2018). Moreover, polymicrogyric (highly folded) cortices tend to be thinner, and lissencephalic (smooth) cortices thicker than typical brains (Llinares-Benadero and Borrell, 2019).

### 4.5. Cortical thickness ratio agrees with predictions from computational models of cortical folding

*In silico* models of the growth and folding of the cortex have revealed fundamental aspects of gyrification, including the consistent placement of certain folds (Tallinen et al., 2016; Toro, 2012; Razavi et al., 2015) and the generation of heterogeneous patterns of stress throughout gyri and sulci (Foubet et al., 2019). These studies have also been extended to comparative neuroanatomy, for instance showing that cortical thickness, surface area, and the degree of folding are related by universal scaling laws that transcend differences among species and individuals (Prothero, 1999; De Lussanet, 2013; Mota and Herculano-Houzel, 2015; Wang et al., 2019). Here, we have looked for the scaling laws that govern the thickness ratio (defined here as the ratio of average thicknesses between convex and concave points). For example, thickness ratio significantly increases with surface area (Fig. 10A), although it has a lower correlation and smaller range (from ~1.05 for the Senegal galago to ~1.3 for humans) than other measures of brain morphology we considered (Fig. 9).

Previous work has shown that patterns of thick gyri and thin sulci naturally emerge through the process of cortical folding (Holland et al., 2018; Wang et al., 2021; Toro and Burnod, 2005). From our previous work, gyral–sulcal thickness differences are predicted to increase with (1) increasing foldedness, (2) increasing cortical thickness, and (3) decreasing gray-white matter stiffness ratio (Holland et al., 2018). While an investigation of tissue properties is outside the scope of this paper, we can test the first two predictions. Indeed, we do see a general increasing trend in the thickness ratio with both average cortical thickness and foldedness (quantified by GI) (Fig. 10B–C). These predictions were based on simulations of homogeneous growth, but preferential growth in gyri and sulci can further affect the evolution of cortical thickness in those regions (Wang et al., 2021).

### 4.6. Cortical thickness varies along a gyral–sulcal spectrum within and across primate species

Our investigation into cortical thickness patterns in 12 primate species shows that the cortex follows similar patterns throughout the folds of each brain and in each species, with thicker convex folds and thinner concave folds. For this study, we used shape index, a dimensionless measure introduced by (Koenderink and van Doorn, 1992) that describes the shape of a surface with a value between −1 and 1. Shape index, assigns a single scalar value to the local shape, passing from cups (which are concave in all orientations) to ruts (concave in one direction and flat in another), to saddles (concave in one direction and convex in another), to ridges (convex in one direction and flat in another), to caps (convex in all orientations) (Fig. 5). Thus, it describes shapes in a smooth spectrum from the most concave to the most convex. This quantity offers advantages over other measures of curvature; for example, the dimensionless nature of shape index makes it ideal for investigating different-sized cortices. Furthermore, only shape index has a bimodal distribution, which provides valuable information about patterns of cortical folding (See Supp. Fig. 1).

Convex shapes are frequently thought of as gyri – and, conversely, concave shapes as sulci – but in reality these are descriptors from two separate domains (Demirci and Holland, 2022). Gyri, sulci, and sulcal walls are anatomical terms that describe gross morphological features of the brain. Concave, convex, and saddle shapes, on the other hand, are geometrical terms that define local shape. Geometry does not correspond exactly to anatomy; while some combinations are more common than others, it is possible to find convex, concave, and saddle shapes in any anatomical category (Demirci and Holland, 2022). For example, in our previous study we showed that there are convex shapes in the depth of sulci that have a higher thickness than the rest of the sulcus (Demirci and Holland, 2022). In this study, we used geometry for the analysis of cortical morphology across a diverse collection of species. This approach does not require detailed segmentation of anatomical gyri and sulci by trained comparative neuroanatomists and/or brain atlases for parcellation of the cortex. By using shape index, we divided the cortex into 9 minor and 3 major shapes. Our findings provide substantial insights into understanding the consistent shape and thickness correlations of the cortex. Similar to our previous study of only humans (Demirci and Holland, 2022), our results here indicate a strong correlation between cortical thickness and shape for each of the species we investigated, despite large variations in gyrification and size of the brains. Our observations showed a well-organized and consistent variation of cortical thickness in a gyral–sulcal spectrum, both within and across species. Specifically, we observed that convex shapes throughout the cortex are consistently the thickest, concave shapes are the thinnest, and saddle shapes lie in the middle (Fig. 5). Additionally, we showed that not only geometry but also the depth of each point consistently correlate with cortical thickness. This local correlation analysis and the calculated Pearson’s *r* values can be found in the supplementary document (Supp. Figs. 4–5).

Moreover, as our previous work has shown that physical forces involved in cortical folding contribute to the pattern of thick peaks and thin valleys, the local shape would likely be a better indicator of the local mechanical state than the neighboring anatomical features.

### 4.7. Physical forces potentially explain consistent variations of cortical thickness

Cortical folds have consistent patterns across individuals of the same population or even across species, as we have shown in this study, which provides evidence for a principal mechanism of folding. We believe that the forces that come into play during folding generate a mechanically favorable state that might also be the most geometrically efficient (Mota et al., 2019). Therefore, cortical folding might represent a physical phenomenon governed by physical and geometrical constraints (Heuer et al., 2019; Budday et al., 2015).

However, it is not plausible to completely ignore the genetic determinants that play a key role in cerebral evolution (Geschwind and Rakic, 2013; Llinares-Benadero and Borrell, 2019), for instance in the difference between gyrencephalic and lissencephalic species. During development, radial glial cells provide a substrate and pathway for neuronal migration from the proliferative ventricular zone towards the cortical plate or pial surface (Rakic and Swaab, 1988; Cowan, 1979). The division of the subventricular zone into inner and outer parts is linked to gyrencephaly of the cortex, with intermediate progenitor cells more abundant in the latter (Reillo et al., 2010). The abundant progenitor cells, associated with genes such as *TRNP1* and *CDH1*, cause further abundance of basal radial glial cells (bRGC) which have basal processes extending to the pial surface but not necessarily to the ventricular surface (Penisson et al., 2019). The abundance of bRGCs causes tangential dispersion through intercalation between classical apical radial glial cells (Reillo et al., 2010), resulting in gyrification (Ronan and Fletcher, 2015). Curved trajectories of bRGCs leads to tangential expansion of the cortical surface area at the expense of a thicker cortex; if the proliferation of bRGCs is reduced, the cortex will be abnormally thick (Reillo et al., 2010). Simply by changing the expression levels of certain genes associated with cellular proliferation, such as *FGF1*, gyrification can be triggered in the normally-lissencephalic mouse cortex (Shinmyo et al., 2022). Therefore, gene expression controls proliferation and migration of neural cells, which trigger tangential expansion, which generates physical forces, which lead to cortical folding.

Tangential expansion is the principal, generalized mechanism of cortical folding (Ronan and Fletcher, 2015; Chavoshnejad et al., 2021). But why are gyri thicker than sulci? When considering the development of heterogeneous thickness patterns, cytoarchitecture may play a role. Cytoarchitectural differences between locations may cause non-uniform tangential expansion (Toro and Burnod, 2005). For example, the patterns of radial glial cells allow greater migration of neurons into gyri (Borrell, 2018; Penisson et al., 2019).

Furthermore, different stresses in gyri and sulci could contribute to thickness differences. Tissue-cut experiments show significant radial axonal tension and compression in developing gyri and sulci, respectively (Xu et al., 2010). Axons are capable of elongating and growing along their longitudinal axis under stretch (Holland et al., 2015) and shrinking when compressed, acting as a viscoelastic solid attempting to maintain a desired level of tension (Bray, 1984; Lamoureux et al., 2010). Axonal elongation in gyri (Holland et al., 2015) could lead to increased thickness, while compression, or shortening, of axons in sulci could contribute to reduced thickness. Mechanical tension is also the regulator for axonal wiring patterns; when there is no tension, e.g. in sulci, neuronal arborization diminishes and the axonal branches retract (Anava et al., 2009), potentially leading to further thinning.

Finally, there is a strong positive correlation between axonal fiber density and cortical thickness (Li et al., 2015). Fiber connections in convex gyri are significantly denser than the ones in concave sulci (Nie et al., 2012; Chavoshnejad et al., 2021), which could further contribute to thickness differences. Moreover, it is well-known that axonal connectivity is more elaborate, and axonal connections more dense, in higher-order species (Chen et al., 2013b; Groden et al., 2020). The increasing density of axonal connections in larger brains might additionally explain the progressive increase in cortical thickness ratio among the primate species we have investigated in this study.

### 4.8. Limitations and further considerations

This is the first comprehensive study that demonstrates the gradual and consistent variation of cortical thickness from sulcal fundi to gyral crowns for 12 different primate species including humans. Despite this significant advance, there are some limitations that will require further efforts to address. First of all, we analyzed only 12 species, three of which (macaques, chimpanzees, and humans) were represented by tens or hundreds of subjects, and the remaining nine species with only a single specimen. Our results are consistent across both the individuals examined and the totality of the species considered. However, in the future, a larger study (considering both more species and more individuals) could prove even more conclusive. Unfortunately, while collaborative data-sharing initiatives for comparative neuroanatomy studies are growing, there are still significant limitations to the availability of non-human subjects, both in terms of the species and the number of specimens of a single species (Neff, 2020). Macaques and chimpanzees are the most abundant species available (Milham et al., 2018; NCBR). While other species can be found, the available images are either postmortem *ex vivo* scans or only a single specimen has been scanned (e.g. Kaas and van Eden, 2011). Similarly, our validation of measurements were limited by the results available in literature (Fig. 4), particularly for less common species.

Moreover, for the species with multiple subjects (macaques, chimpanzees, and humans) their ages span a wide range. Some of the subjects in this study are not yet adults while others would be considered by primatologists as senile. For example, macaques reach puberty around 2.5 to 4.5 years, sexual maturity at 3 years, and adulthood at 8 years. Average cortical thickness decreases with age and cortical atrophy is present during normal aging for humans (Hurtz et al., 2014; Fjell et al., 2015; Minkova et al., 2017), rhesus macaques (Koo et al., 2012), and chimpanzees (Vickery et al., 2020). Our age and atrophy analysis include only humans and chimpanzees but not rhesus macaques, due to limited public information of age of rhesus macaques during scanning. However, earlier studies strongly suggest cortical atrophy of rhesus macaques with respect to aging (Amlien et al., 2016; Chen et al., 2013a)

Besides age differences, we note that some species were imaged postmortem, while the rest of the species (rhesus macaque, chimpanzee, bonobo, gorilla, and human) were imaged *in vivo*. By collecting data from both types of species we aimed to maximize our number of species and the range of brain sizes and forms. Unfortunately, the brain is known to reduce its volume *ex vivo* due to prolonged hours of formalin fixation (Calabrese et al., 2015). For instance, one study found a volume reduction of 3.3% after a 70-day fixation in 10% formalin (Schulz et al., 2011). This volume reduction might help explain why we observed a slightly thicker cortex for rhesus macaques compared to their neighbors (Fig. 6), as rhesus macaques are imaged *in vivo* while the others were imaged postmortem.

Secondly, we obtained MRIs of various non-human primate species (macaques, chimpanzees, bonobo, and gorilla) from different public resources. Public repositories are very valuable in that they allow many researchers from outside the imaging community to access and utilize those resources in their studies. However, while minimal data standardization practices have been established for human imaging in order to enable large collaborative projects between laboratories, nothing similar has been agreed upon for non-human primate imaging research around the world. Different laboratories often use different protocols, with different image acquisition parameters and image resolutions (Table 1, Table 2) (Autio et al., 2021) (see Section 1). Recently, minimum specifications were recommended for non-human primate imaging studies, in order to improve robustness and reproducibility of studies (Autio et al., 2021), including a minimum scanner strength of 3T. Unfortunately, some of our species (bonobo and gorilla) were acquired at 1.5T, which can lead to segmentation errors and affect the reconstructed surface quality.

Standardization and harmonization of non-human primate data acquisition and imaging protocols are important for comparative non-human primate studies but also challenging due to necessary customization of hardware to compensate for differences in head size and improve signal-to-noise ratio (Hayashi et al., 2021; Autio et al., 2021; Pomponio et al., 2020; Ose et al., 2022). One solution for harmonized data acquisition is to adjust the voxel resolution to the thinnest parts of the cortex to alleviate partial volume effects and harmonize the differences, for example in cortical thickness across species (Hayashi et al., 2021), but this requires development of species-specific receiver coils (Autio et al., 2020).

Another approach, often used in studies of human subjects, involves advanced statistical methods to remove sources of variability, including data acquisition protocols and hardware, from multi-site large datasets (Pomponio et al., 2020; Koike et al., 2021). These statistical approaches include adjustment of mean and variance of imaging measurements across sites for robust predictions (Pomponio et al., 2020) and the traveling subject approach, in which multiple subjects travel to multiple sites to estimate and quantify the variability between sites (Koike et al., 2021; Yamashita et al., 2019). We did not perform statistical data harmonization in this study, as harmonization protocols are most relevant for big (>1k) neuroimaging multi-site studies. Here, the only species that were imaged at multiple sites were humans and macaques. For humans, we relied on existing standardization and quality analysis of the ABIDE project (Pardoe et al., 2016). For macaques, only certain sites were included based on similarity in subjects, imaging hardware, and protocols. In the future, standardization of data acquisition protocols for non-human primate imaging datasets, potentially via a variation of the traveling subject approach, would be invaluable assets for multi-site comparative neuroanatomy imaging studies.

In general, estimation of surface measures (cortical thickness, sulcal depth, curvature, shape index, etc.) depends on data acquisition parameters, image resolution, and the accuracy of cortical surface reconstructions. The differences between the image acquisition parameters, resolution, and the processing pipelines might develop slight variations between measures for the same subject, which would be hard to detect unless an exactly similar method (for both image acquisition and processing) is employed (Dias et al., 2022; Velázquez et al., 2021).

Third, we adopted species-specific solutions for preprocessing MR images. While human MR images are commonly preprocessed by benchmarked pipelines such as Freesurfer (https://surfer.nmr.mgh.harvard.edu), there is no consensus as to the optimal tool to preprocess MR images of various non-human primate species. Therefore, custom preprocessing pipelines are developed to tackle size, form, and tissue contrast differences between species.

These pipelines are mostly species-specific, hindering their use for other species, and highly customized, creating a barrier to reproducibility. In the future, a more general, one-size-fits-all preprocessing pipeline – from skull-stripping to surface generation – could potentially address these challenges and enable the expansion of this study to additional species with MR images but no population template available.

In general, the biggest limitation of this study is this non-unified approach, from the image acquisition using different parameters (Table 2), to the sourcing of images from various public resources, to the different processing approaches that were unique for each species (Table 1). Therefore, although in some senses this non-unified approach is a weakness of the study, it also increases our confidence in our results. Although we collected the images from different resources and employed different processing tools, our findings point us to the consistent structure of the cortex; there is strong correlation between thickness and shape in each of our 12 species.

Finally, cortical thickness, sulcal depth, and gyrification are known to vary in different regions of the brain. Similar to most comparative studies (e.g. Hofman, 1985), we presented our allometric scaling findings based on global averages considering the whole brain (Fig. 9). However, we predict that more interesting results could be found by performing regional comparisons between corresponding areas of brains of different primate species.

## 5. Conclusion

In summary, this study demonstrates that consistent cortical thickness patterns can be found across 12 different primate species with varying forms and sizes. Similar to human brains (Demirci and Holland, 2022), cortical thickness patterns are strongly consistent in non-human primates. For each species, the cortex is thickest at convex points (generally gyri), thinner at saddle shapes (generally lateral bends in sulcal walls) and thinnest at concave shapes (generally sulci). Furthermore, the variation of cortical thickness and folding patterns (folding amplitude and gyrification) are strongly correlated with size, which recapitulates the mathematical models developed in previous studies that simulate growth and development of the cortex.

Because of this, we hypothesize that the mechanical forces generated during growth and development of the cortex strongly affect the resulting cortical morphology and its relationship with cortical thickness through a principal mechanism of cortical folding. While there are likely genetic and other factors that also influence cortical thickness (Wang et al., 2021), they seem to work alongside mechanical forces to produce consistent cortical thickness patterns both within and between species (Van Essen et al., 2018). This interpretation implies that patterns of cortical thickness are a natural consequence of cortical folding rather than the cause (Mota and Herculano-Houzel, 2012), although mechanical models and computer simulations show that variations in initial thickness strongly affect the bending resistance of the cortex (Holland et al., 2018; Toro and Burnod, 2005; Tallinen et al., 2014).

To the best of our knowledge, this is the first comprehensive study of local cortical thickness variations in non-human primate species. Our results demonstrate the gradual and consistent variation of cortical thickness from sulcal fundi to gyral crowns for 12 different primate species including humans. Our findings provide more insight into our understanding of well-characterized patterns of the cortex. We also hypothesize that our findings might hold across mammalia. In order to test this, future work should expand on this study to include more mammalian species. Additionally, questions remain about the functional implications of these cortical thickness patterns, which could potentially shed light on cases of disordered folding in humans. To encourage future contributions to these topics from other researchers in diverse disciplines, we have made all of the surfaces, scripts, and data from this work publicly available.

## Supplementary Material

1

## Figures and Tables

**Table 1:** Primate species used in this study along with their total number subjects, age-span, and status during scan. Scanning parameters for each species, processing tool(s), and resources are also listed. All images are T1-weighted. (PM: Postmortem, IV: In-vivo, NCBR: National Chimpanzee Brain Resource, PBB: Primate Brain Bank, CAT: Computational Anatomy Toolbox, SPM: Statistical Parametric Mapping, PRIME-DE: Primate Data Exchange, *See Demirci and Holland (2022) for details).

Common name	Scientific name	N	Gender	Age	Subject	Scan parameters		Processing	Source
			M:F	[years]	Status	Strength	Voxel size [mm^3^]	Tools(s)	
Senegal galago	*Galago senegalensis*	1	M	20.9	PM	7T	0.3 × 0.3 × 0.3	Freesurfer	PBB
Night monkey	*Aotus lemurinus*	1	M	15.3	PM	7T	0.4 × 0.4 × 0.4	Freesurfer	PBB
White-faced saki	*Pithecia pithecia*	1	M	4.0	PM	7T	0.6 × 0.6 × 0.3	Freesurfer	PBB
Tufted capuchin	*Sapajus apella*	1	M	22.0	PM	7T	0.6 × 0.6 × 0.6	Freesurfer	PBB
Rhesus macaque	*Macaca mulatta*	31	29:2	2.7–8.0	IV	3T	See Table 2	ANTs, AFN	PRIME-DE
Black-white colobus	*Colobus guereza*	1	M	23.7	PM	7T	0.6 × 0.6 × 0.6	Freesurfer	PBB
Wooly monkey	*Lagothrix lagotricha*	1	F	8.0	PM	7T	0.6 × 0.6 × 0.6	Freesurfer	PBB
Gray-cheeked mangabey	*Lophocebus albigena*	1	F	27.0	PM	7T	0.6 × 0.6 × 0.6	Freesurfer	PBB
Chimpanzee	*Pan troglodytes*	54	19:35	8.0–53.0	IV	3T	0.6 × 0.6 × 0.6	CAT, SPM	NCBR
Bonobo	*Pan paniscus*	1	M	8.0	IV	1.5T	0.7 × 0.7 × 0.7	BrainVisa	NCBR
Gorilla	*Gorilla gorilla*	1	M	8.0	IV	1.5T	0.7 × 0.7 × 0.7	BrainVisa	NCBR
Human	*Homo Sapiens*	501	414:87	7.0–56.0	IV	3T	Varies per site*	Freesurfer	ABIDE-I

**Table 2:** PRIMatE Data Exchange (PRIME-DE) MRI×data ×acquisition details of rhesus macaque species for each site. (SP: Siemens Prisma, STT: Siemens Tim Trio).

Site	N	Voxel size [mm^3^]	Scan parameters				Scanner
			TR	TE	TI	Flip angle	
			[ms]	[ms]	[ms]	[°]	
AMU	4	0.8 × 0.8 × 0.8	2900	2.04	1000	8	SP
ECNU(C)	10	0.75 × 0.75 × 0.8*	2200	2.69–3.71	–	7–9	STT
ECNU(K)	4	0.8 × 0.8 × 1.0	3000	77.00	900	15	STT
ION	4	0.5 × 0.5 × 0.5	2500	3.12	1100	9	STT
NKI	2	0.5 × 0.5 × 0.5	2500	3.87	1200	8	STT
Princeton	2	0.5 × 0.5 × 0.5	2700	2.32	850	9	SP
Rockefeller	5	0.5 × 0.5 × 0.5	2300	2.95	1100	8	STT

* Resolution was listed as 0.6 × 0.6 × 0.6, 0.75 × 0.75 × 0.8.

**Table 3:** Sources for cortical thickness values used for comparison in Fig. 4. Global data (●) were collected when available; if not, regional data (▲) were averaged to get a global estimate. When neither were available, a methodological comparison was conducted comparing results for the same specimen from our computational pipeline to Freesurfer’s surface-based morphometry approach (■).

	Species	Source
■	Senegal galago	Bryant et al. (2021)
■	Night monkey	Bryant et al. (2021)
■	White-faced saki	Bryant et al. (2021)
●	Tufted capuchin	Herculano-Houzel et al. (2008), Herculano-Houzel (2015)
●	Rhesus macaque	Hofman (1985), Bourgeois et al. (1994), Koo et al. (2012), Herculano-Houzel (2015), Lepage et al. (2021), Hayashi et al. (2021)
■	Black-white colobus	Bryant et al. (2021)
■	Wooly monkey	Bryant et al. (2021)
■	Gray-cheeked mangabey	Bryant et al. (2021)
●	Chimpanzee	Donahue et al. (2018), Xiang et al. (2020), Autrey et al. (2014), Hopkins et al. (2019)
▲	Bonobo	Hopkins et al. (2015)
▲	Gorilla	Hutsler et al. (2005)
●	Human	Hofman (1985), Herculano-Houzel (2015), Donahue et al. (2018), Xiang et al. (2020), Fischl and Dale (2000), Hurtz et al. (2014)

## Data Availability

The human imaging data used in the preparation of this article was obtained from the preprocessed neuroimaging data of Autism Brain Imaging Data Exchange I (ABIDE I) repository which is an international neuroimaging data-sharing initiative. The data was obtained from the website https://fcon_1000.projects.nitrc.org/indi/abide/abide_I.html. The preprocessed surfaces was obtained from http://preprocessed-connectomes-project.org/abide/ (Cameron et al. 2013). The macaque imaging data was obtained from the publicly available PRIMatE Data Exchange (PRIME-DE) repository. The data was obtained from the website https://fcon_1000.projects.nitrc.org/indi/indiPRIME.html. We used publicly available codes to process the rhesus macaque imaging data (https://github.com/jms290/NMT/tree/master/NMT_v1.3). The chimpanzee, bonobo, and gorilla imaging data was obtained from the publicly available National Chimpanzee Brain Resource (NCBR) repository. The data was obtained from the website https://www.chimpanzeebrain.org. We used publicly available codes to process the chimpanzee imaging data (https://github.com/viko18/JunaChimp). The preprocessed surfaces for the remaining 7 species was obtained from doi.org/10.5281/ZENODO.5044936. All further analyses and visualization were performed by our publicly available computational pipeline (https://github.com/mholla/curveball). All scripts generated for this study, including code sufficient to reproduce all figures, are available at https://github.com/mholla/NIMG23. Additionally, the data for all 595 subjects of all 12 species are available at doi.org/10.5281/zenodo.7574350, including the pial, white, and alpha surfaces, as well as cortical thickness, area, sulcal depth, and curvature at each vertex.
