# Supplementary material for: Systematic cortical thickness and curvature patterns in primates
Source: Neuroimage. Author manuscript; Available in PMC 2023 Sep 1. (PMC10443624; doi:10.1016/j.neuroimage.2023.120283)
Supplement: 1 [file NIHMS1924378-supplement-1.pdf]

Species	Number of vertices	Mesh resolution [mm]	Alpha value
Senegal galago	∼20k	0.3	4
Night Monkey	∼20k	0.6	5
White-faced saki	∼20k	0.7	6
Tufted capuchin	∼25k	0.9	9
Rhesus macaque	∼25k	1.1	9
Black-white colobus	∼25k	1.2	9
Wooly monkey	∼25k	1.0	10
Gray-cheeked mangabey	∼25k	1.1	10
Chimpanzee	∼35k	1.5	10
Bonobo	∼25k	1.7	10
Gorilla	∼25k	1.7	10
Human	∼40-45k	2.2	20

Table 1: Mesh properties for each species

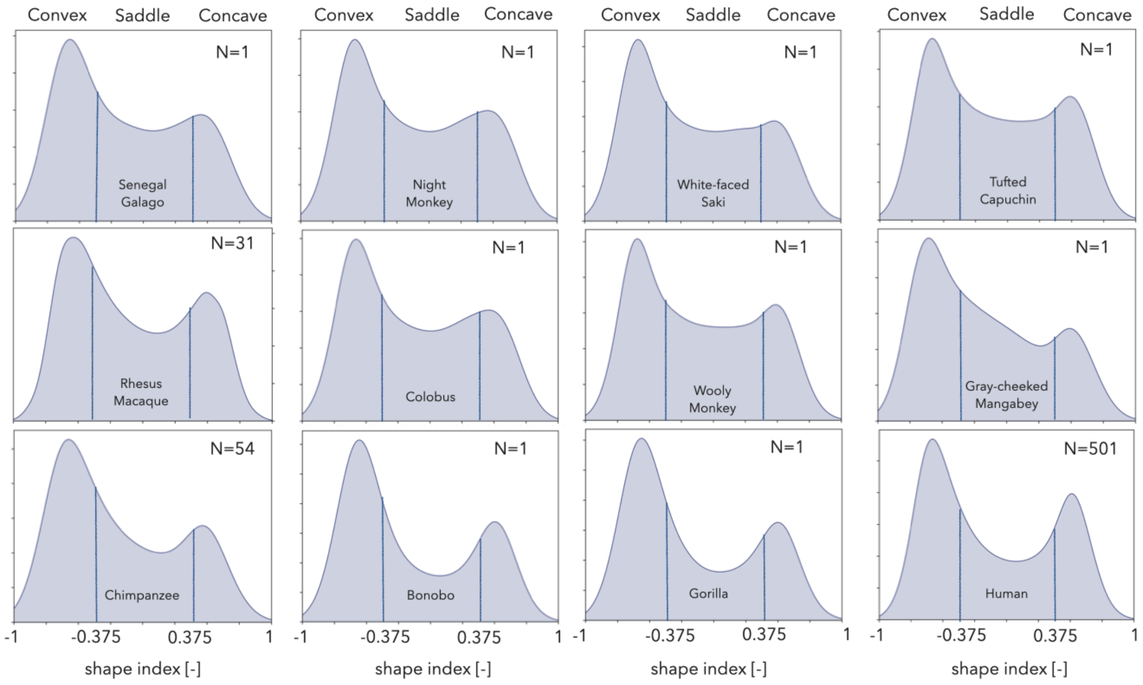

Figure 1: Distribution of shape index for all species. Shape index value of [-1, -0.375] are convex, [0.375, 1] are concave, and [-0.375, 0.375] are classified as saddle. As size of the brain increases for each species, degree of foldedness also increases, which leads to sulcal invaginations and increase in the frequency of concave points.

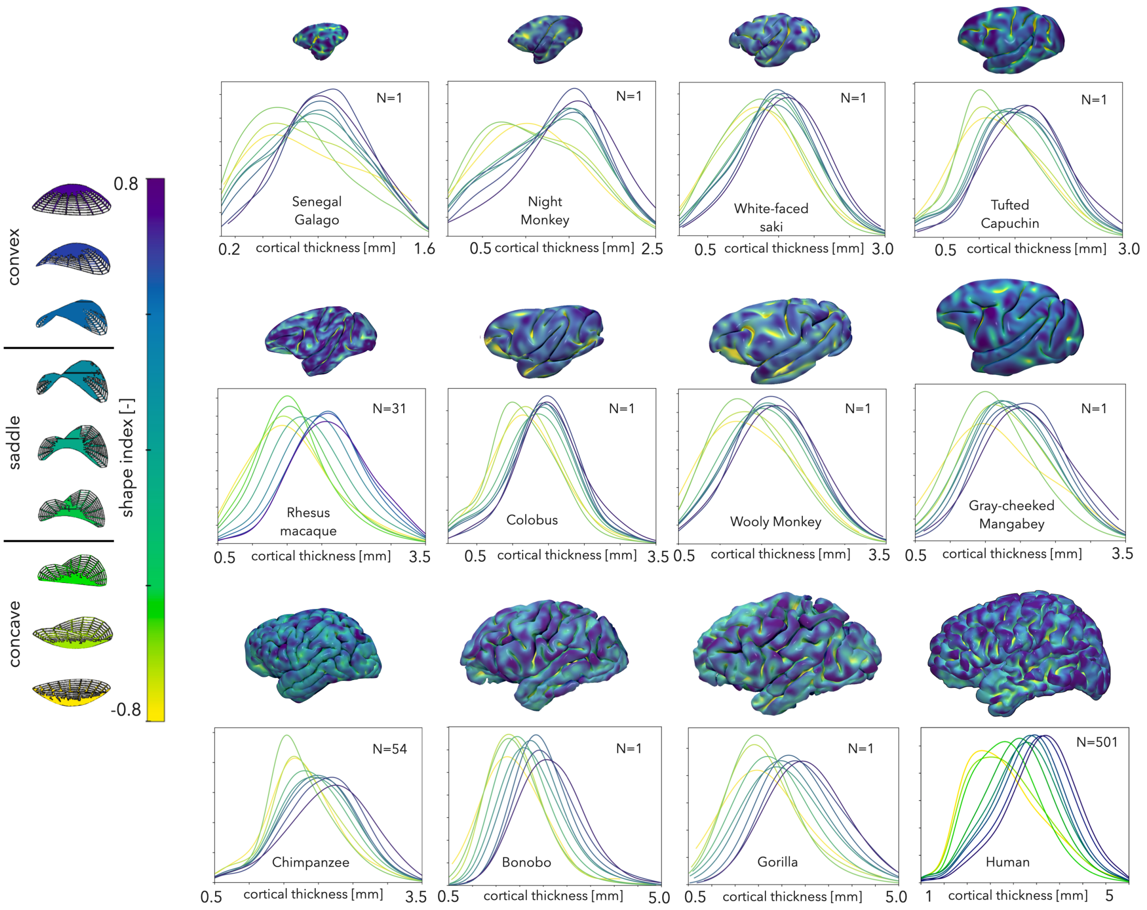

Figure 2: Distribution of cortical thickness with respect to shape for all species. For each species, cortical thickness varies gradually from thinnest concave shapes to thickest convex shapes.

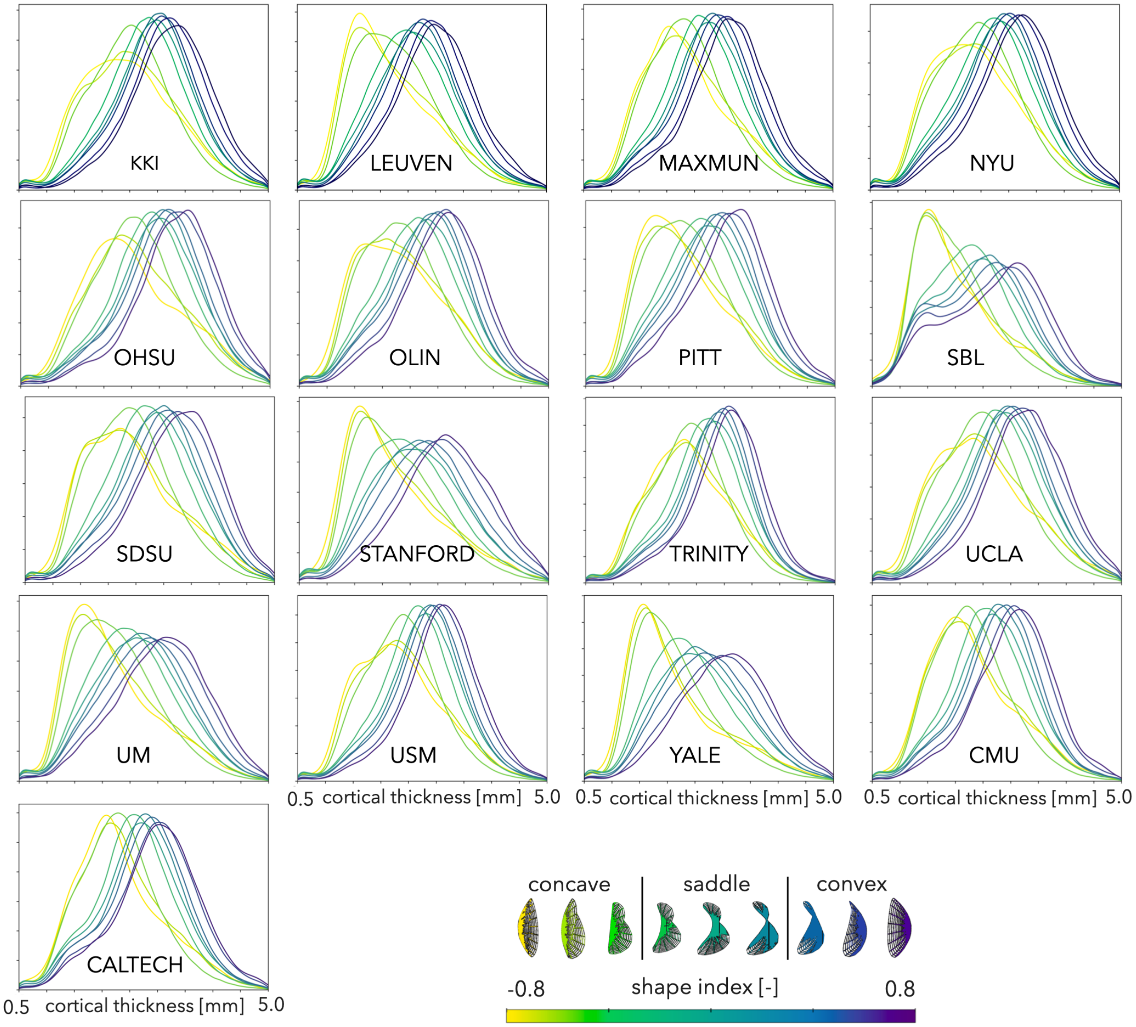

Figure 3: Systematic correlation of cortical thickness with shape for each subset of the ABIDE dataset.

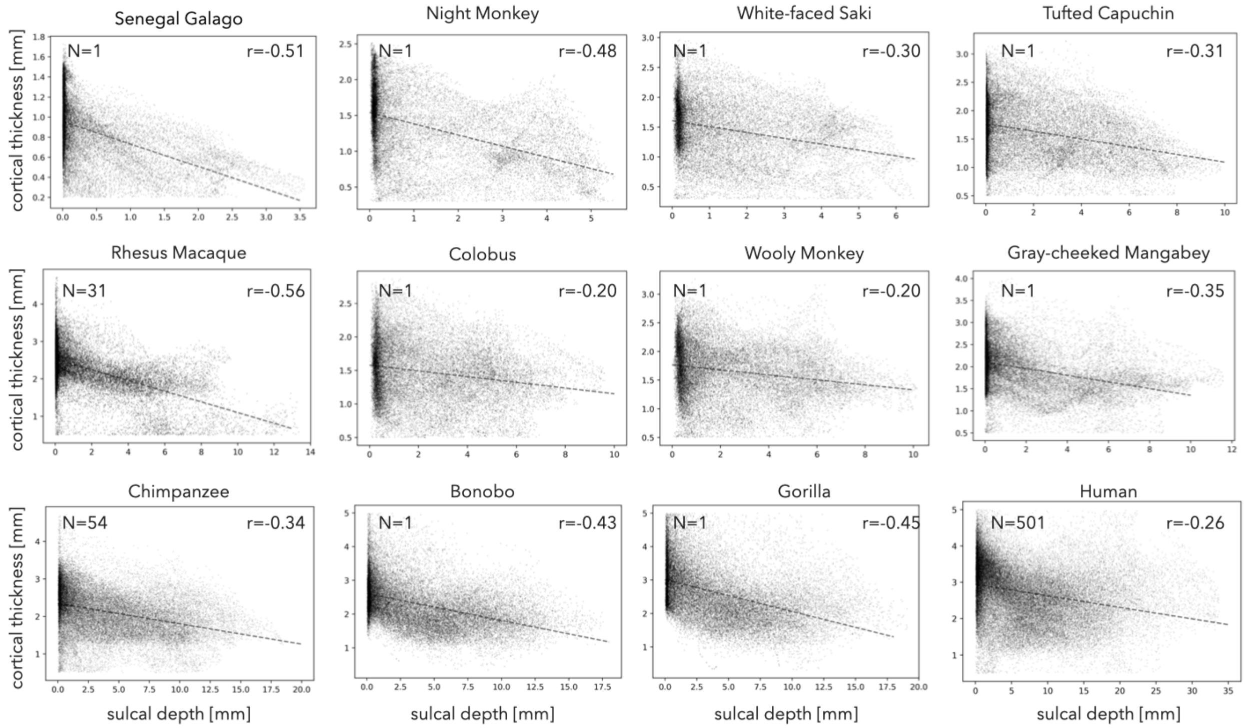

Figure 4: Systematic correlation of local cortical thickness with local sulcal depth for each species. The local data represent one hemisphere of a single subject. Pearson's r values are shown.

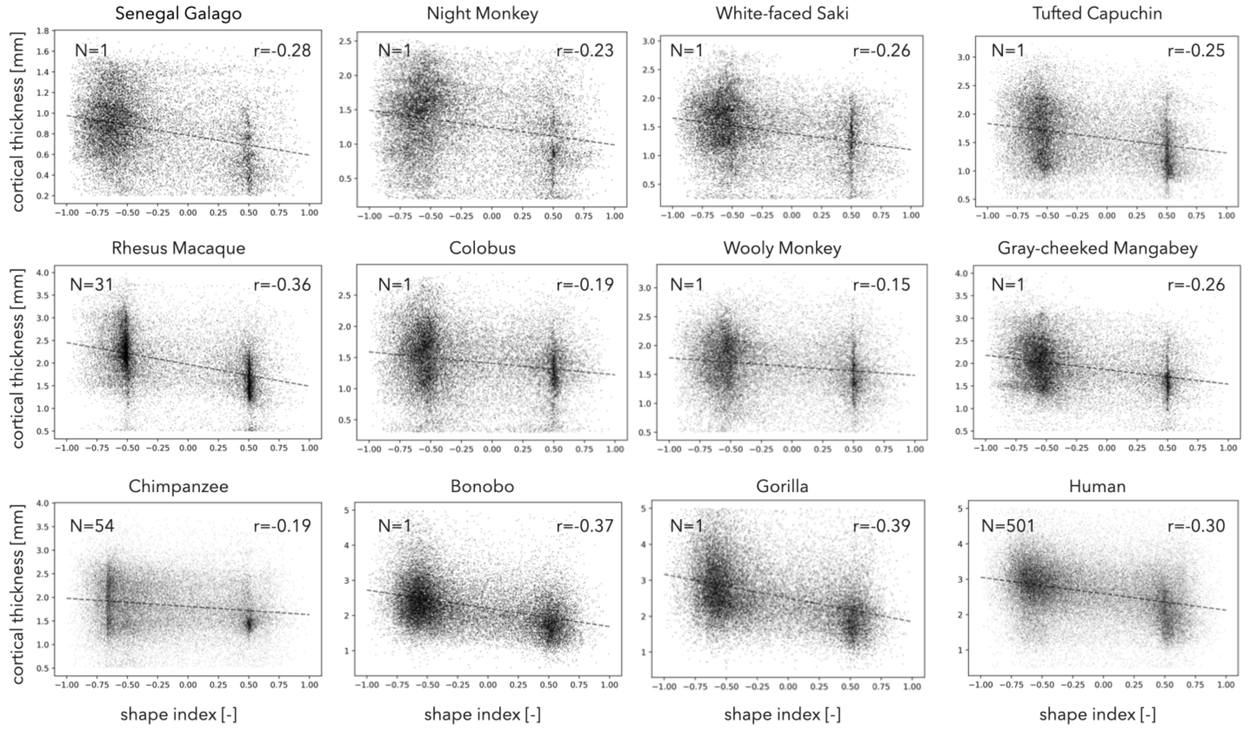

Figure 5: Systematic correlation of local cortical thickness with local shape index for each species. The local data represent one hemisphere of a single subject. Pearson's r values are shown.
